# Supplementary material for: Synthesis and Pesticidal Activity of New Niacinamide Derivatives Containing a Flexible, Chiral Chain
Source: Molecules. 2022 Dec 21;28(1):47. doi: 10.3390/molecules28010047 (PMC9822348; doi:10.3390/molecules28010047)
Supplement: Supplementary file 1 [file molecules-28-00047-s001.zip › molecules-2060161-supplementary.pdf]

Supplemental Materials

Synthesis and Pesticidal Activity of New Niacinamide Derivatives Containing a Flexible, Chiral Chain

Zhe-Cheng Wei[1], Qiao Wang[1], Li-Jing Min[2], Joanna Bajsa-Hirschel[3], Charles L. Cantrell[3], Liang Han[2], Cheng-Xia Tan[1], Jian-Quan Weng[1], Yu-Xin Li[6], Na-Bo Sun[5]*, Stephen O. Duke[4]*, Xing-Hai Liu[1]*

[1] College of Chemical Engineering, Zhejiang University of Technology, Hangzhou, 310014, Zhejiang, China; 398845586@qq.com(Z.W.); 751716045@qq.com(Q.W.);

[2] College of Life Science, Key Laboratory of Vector Biology and Pathogen Control of Zhejiang Province, Huzhou University, Huzhou, 313000, Zhejiang, China, 178341894@qq.com(L.J.)

[3] Natural Products Utilization Research Unit, Agricultural Research Service, U.S. Department of Agriculture, P.O. Box 1848, University, Mississippi 38677, USA, charles.cantrell@usda.gov(C.L.); joanna.bajsa-hirsche@usda.gov(J.B.)

[4] National Center for Natural Product Research, School of Pharmacy, University of Mississippi, P.O. Box 1848, University, Mississippi, 38677, USA, sduke@olemiss.edu(S.D.)

[5] College of Biology and Environmental Engineering, Zhejiang Shuren University, Hangzhou 310015, Zhejiang, China, nabosun@126.com(N.B.)

[6] State Key Laboratory of Elemento-Organic Chemistry, Department of Chemistry, Nankai University, Tianjin, China, Key Laboratory of Study and Discovery of Small Targeted Molecules of Hunan Province, Hunan Normal University, Changsha, Hunan, China, liyx128@nankai.edu.cn(Y.X.)

Table of contents

1. The spectrocopy of target compounds………...................... 2-10

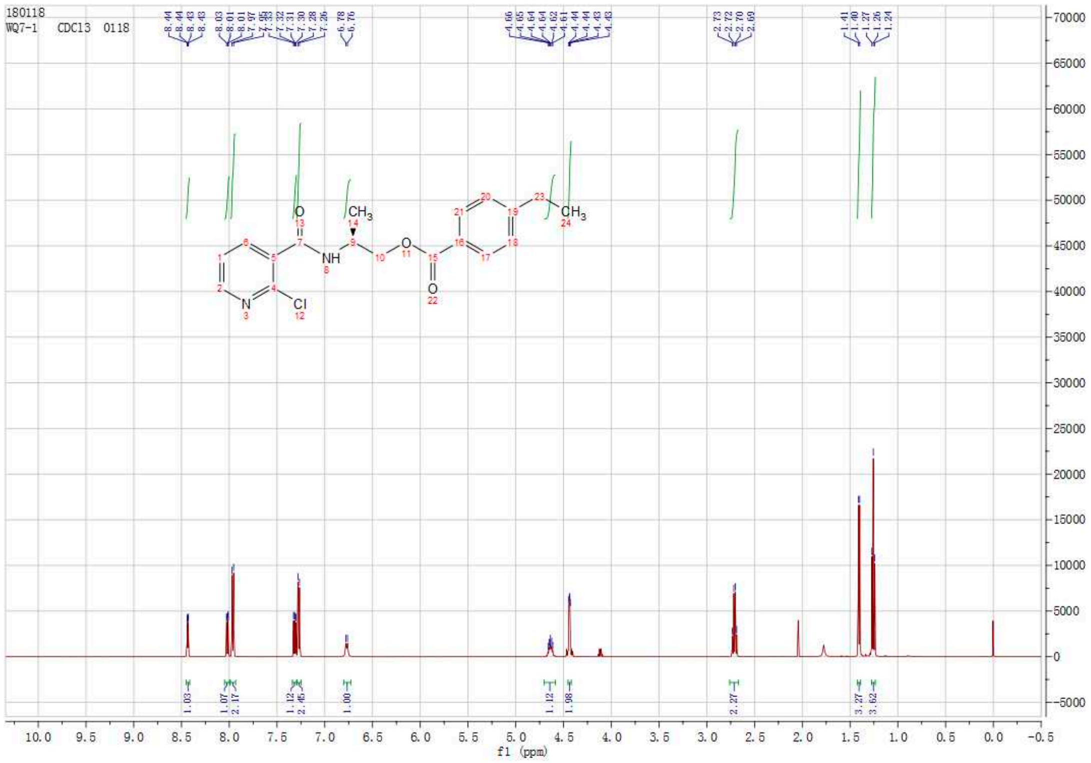

^{1}H NMR of compound 3a

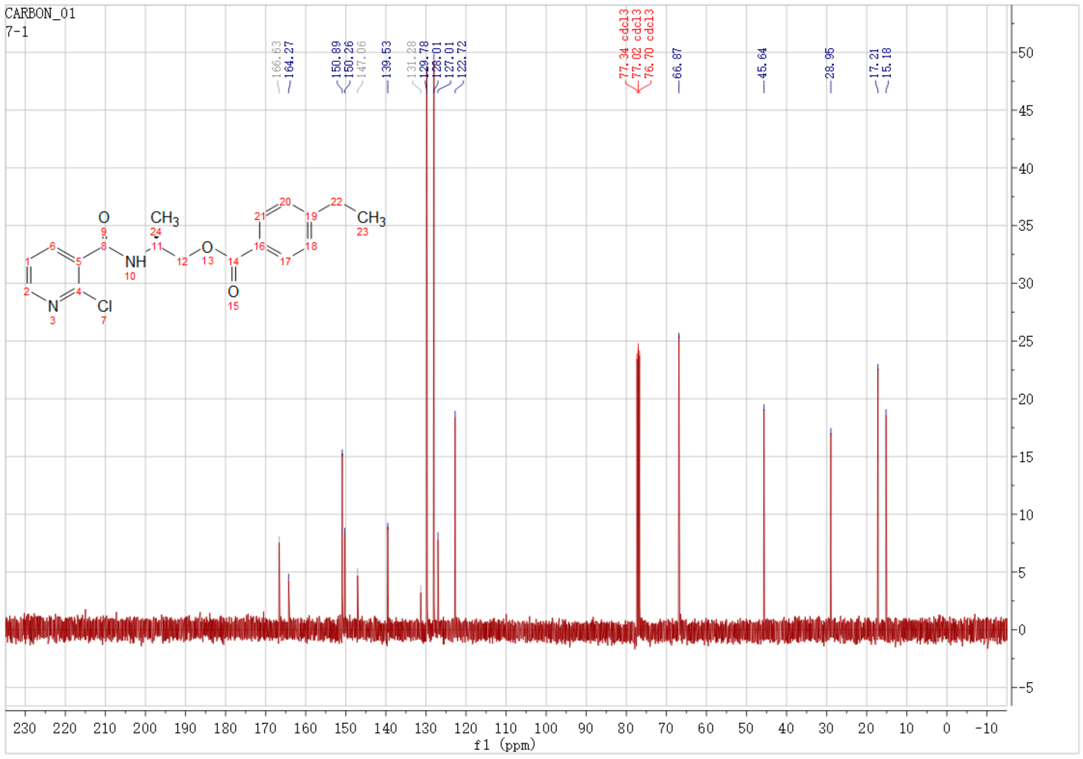

^{13}C NMR of compound 3a

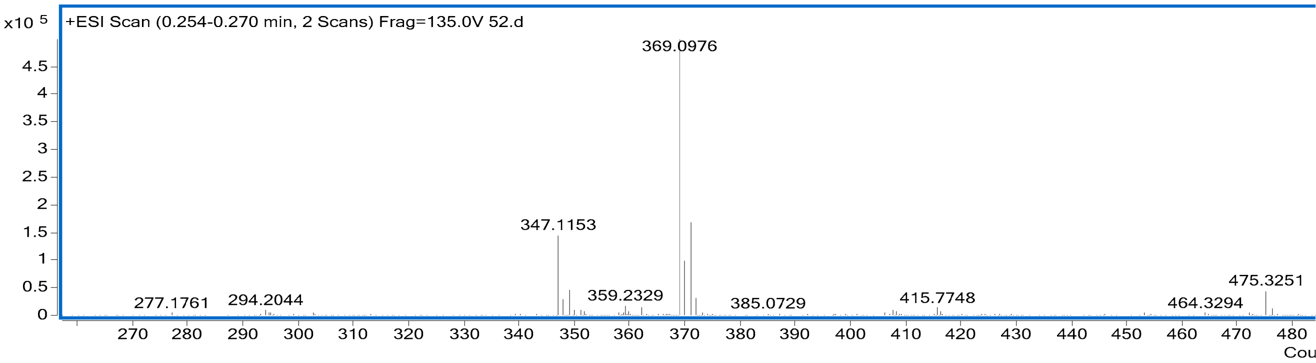

HRMS of compound 3a

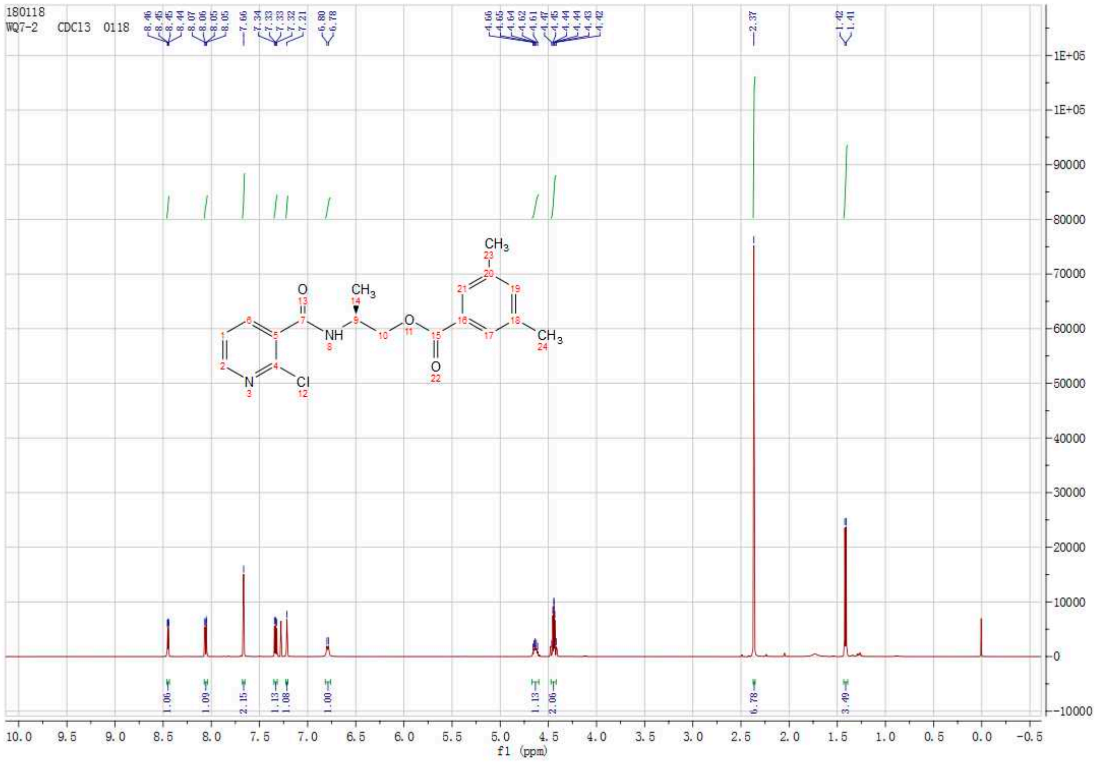

^{1}H NMR of compound 3b

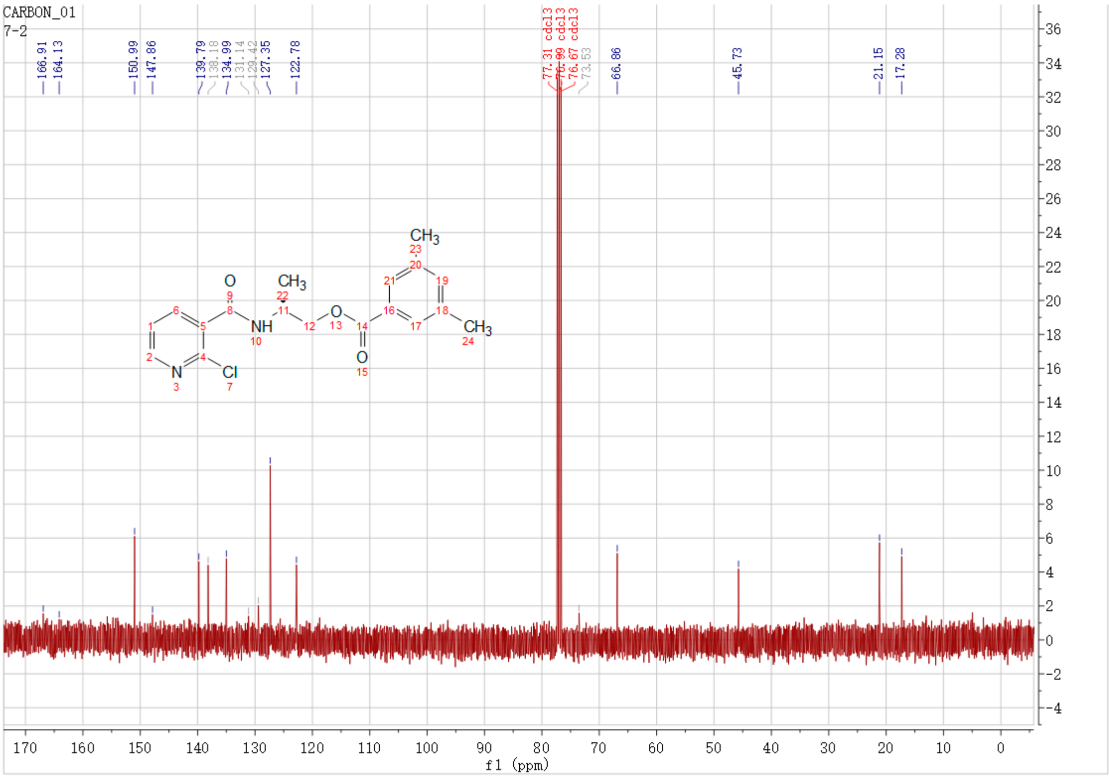

^{13}C NMR of compound 3b

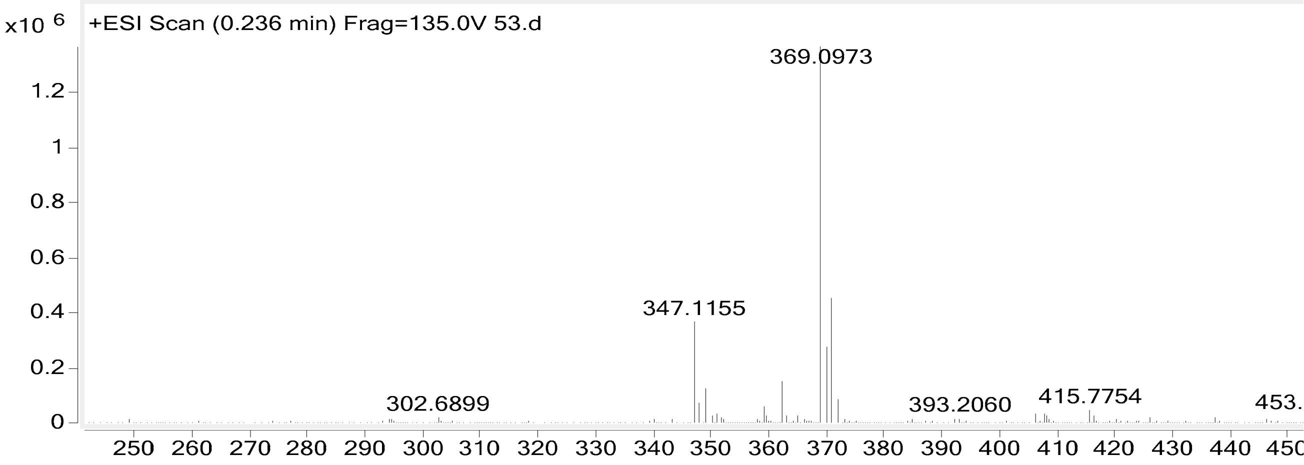

HRMS of compound 3b

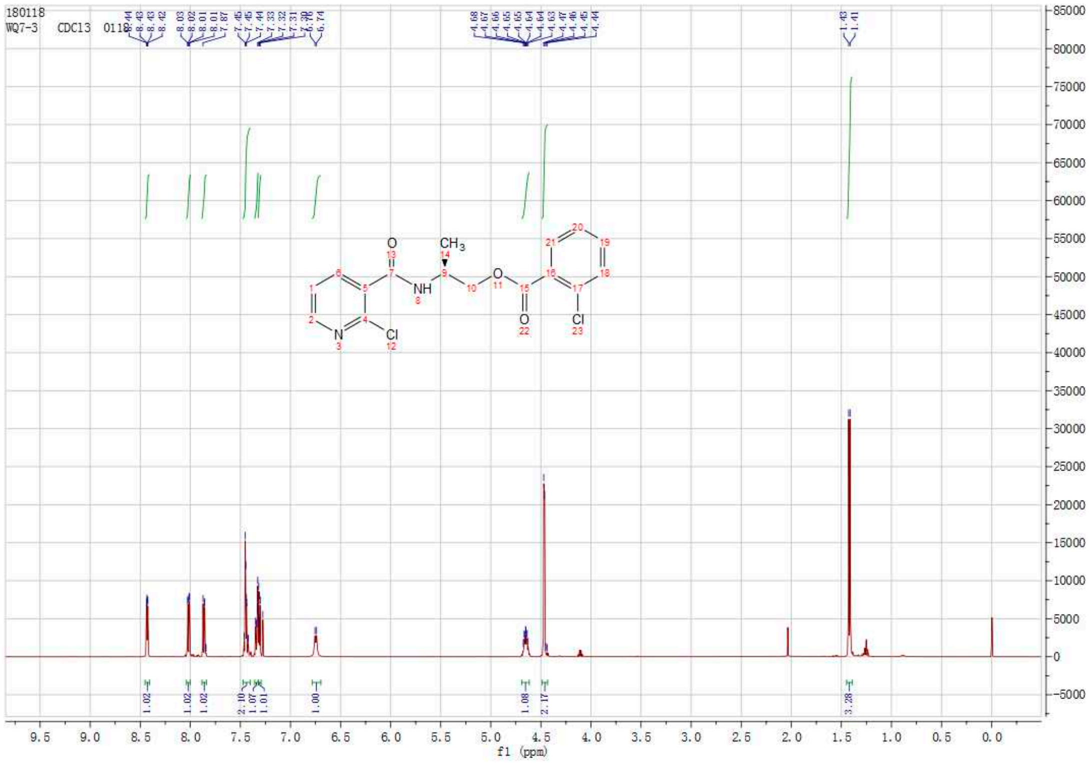

^{1}H NMR of compound 3c

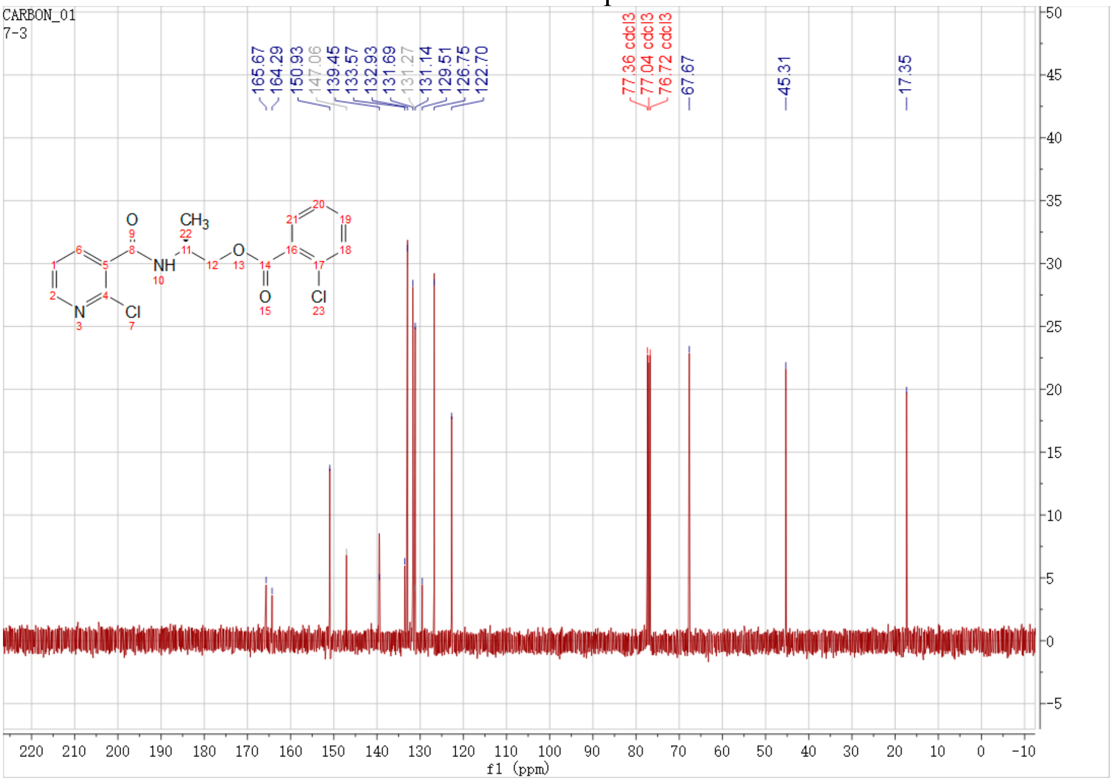

^{13}C NMR of compound 3c

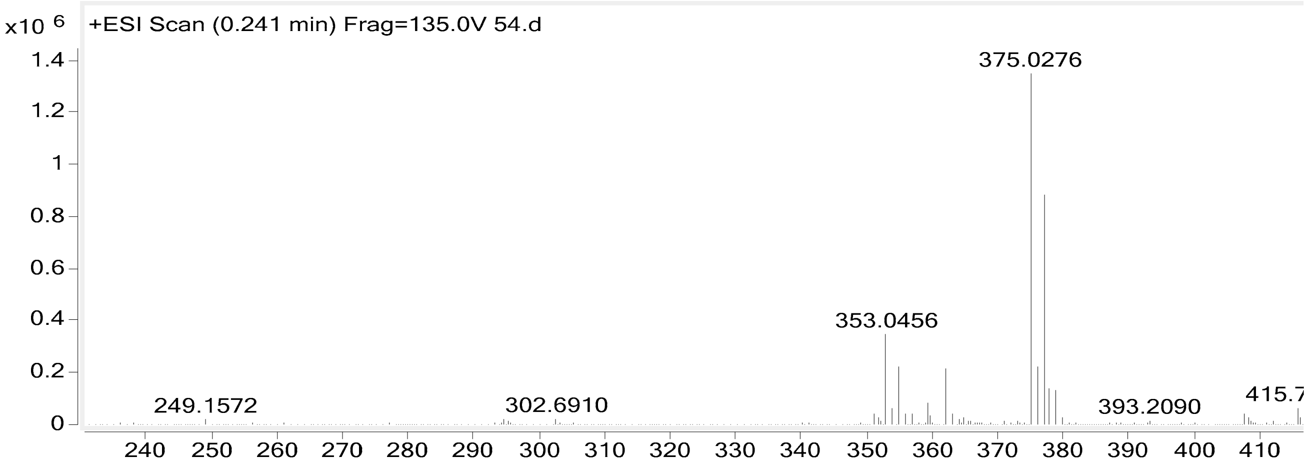

HRMS of compound 3c

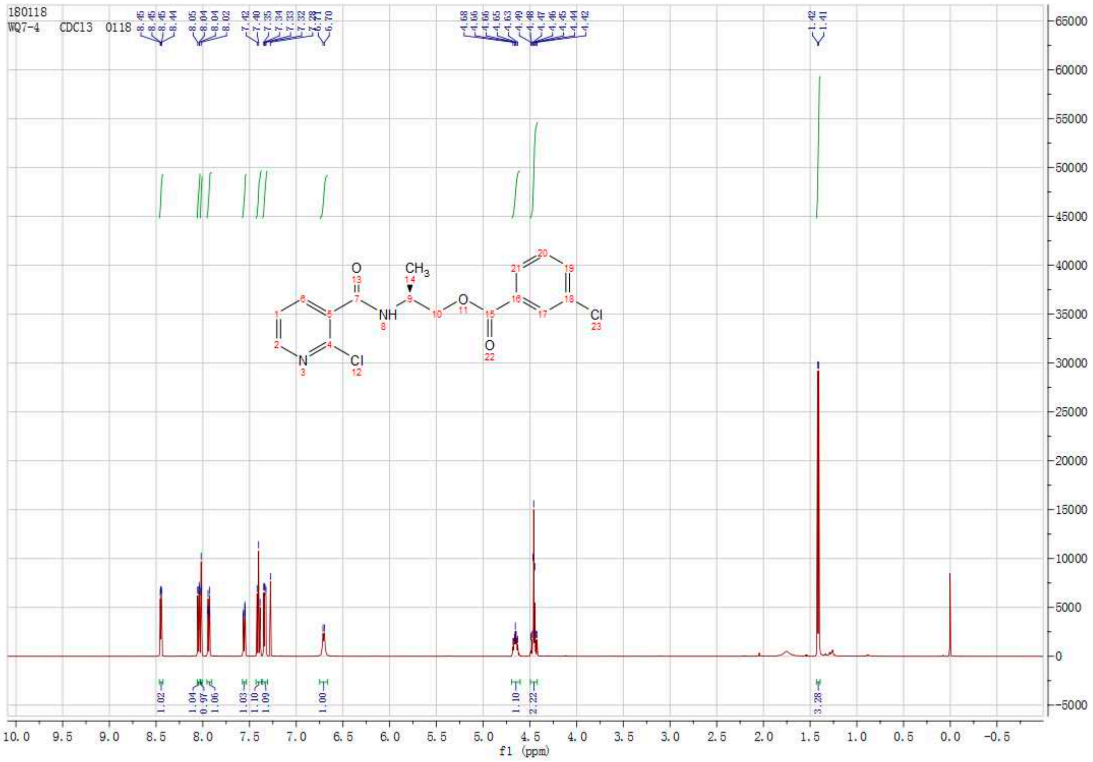

^{1}H NMR of compound 3d

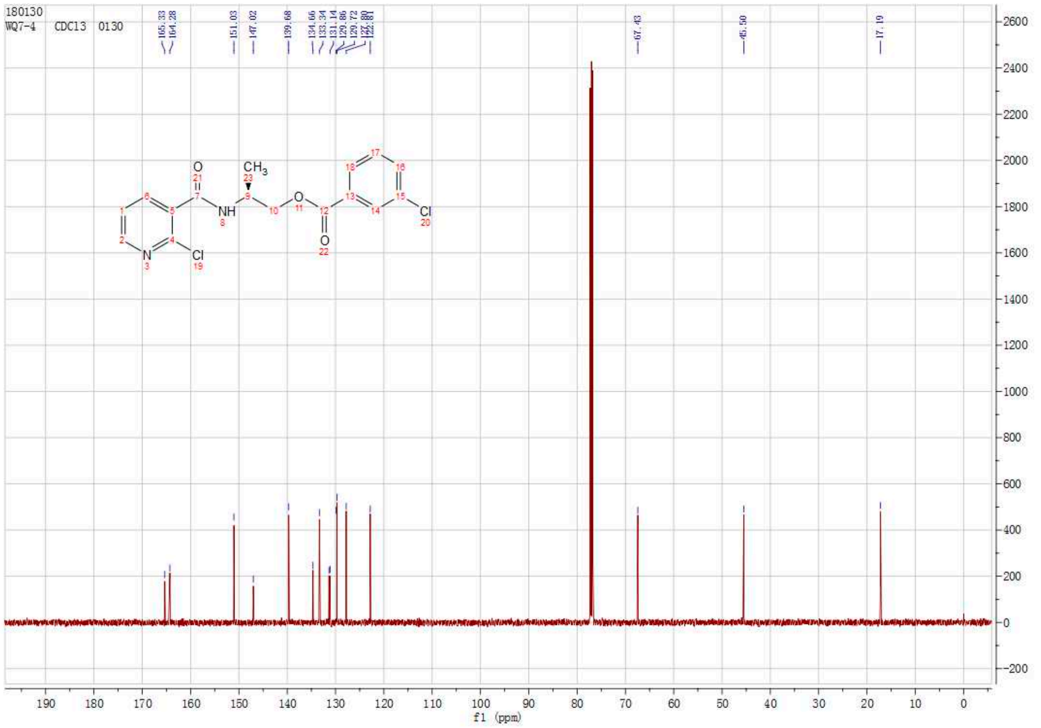

^{13}C NMR of compound 3d

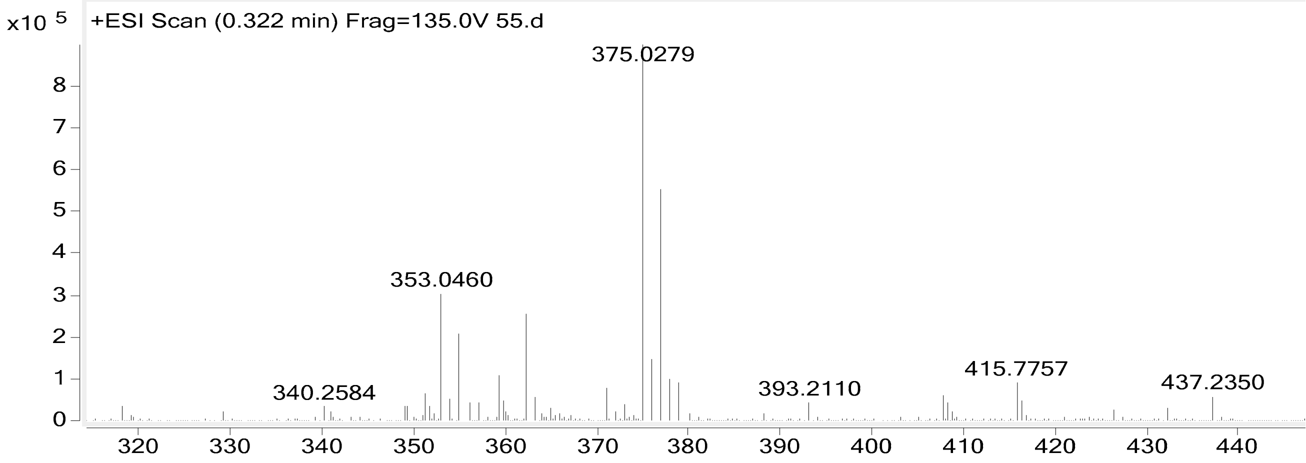

HRMS of compound 3d

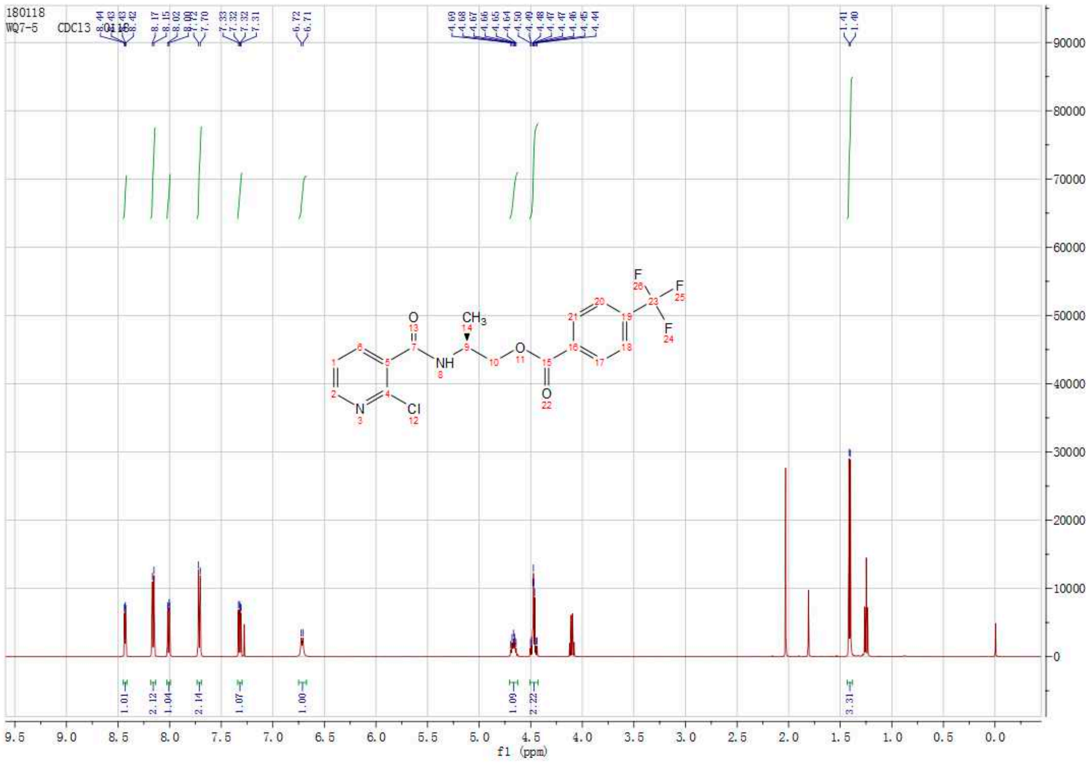

^{1}H NMR of compound 3e

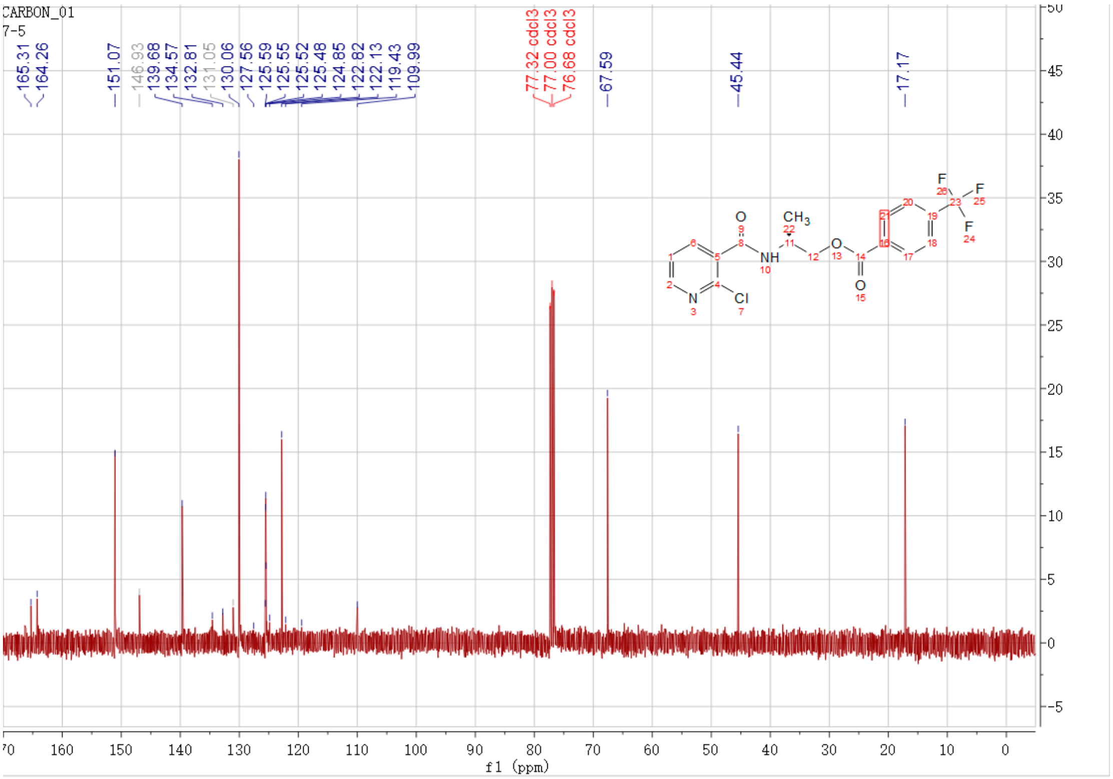

^{13}C NMR of compound 3e

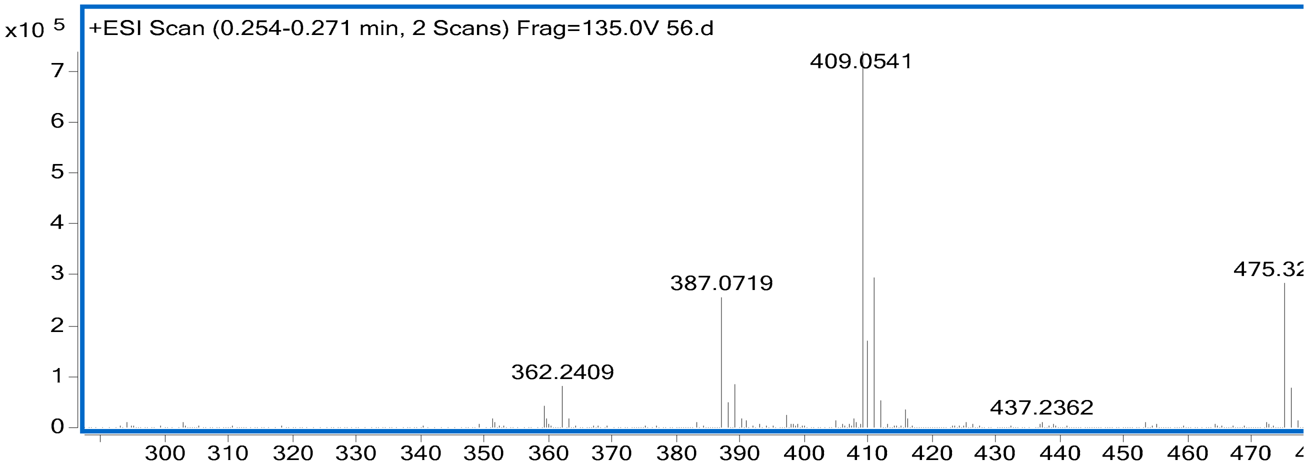

HRMS of compound 3e

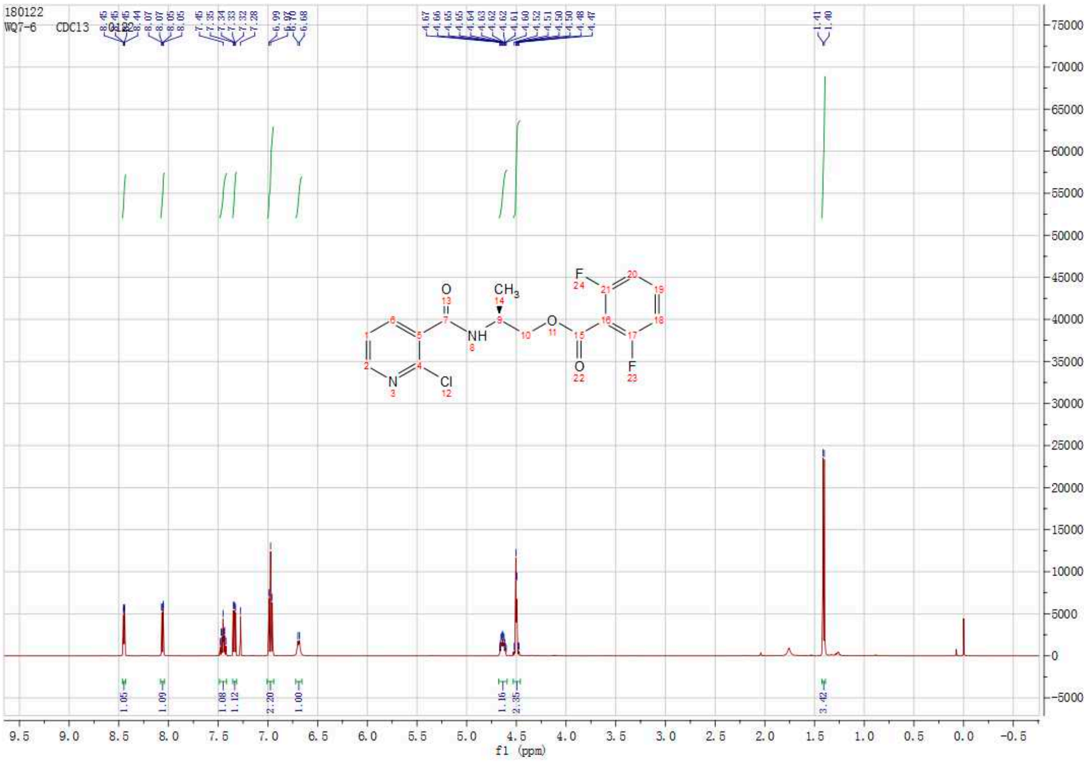

^{1}H NMR of compound 3f

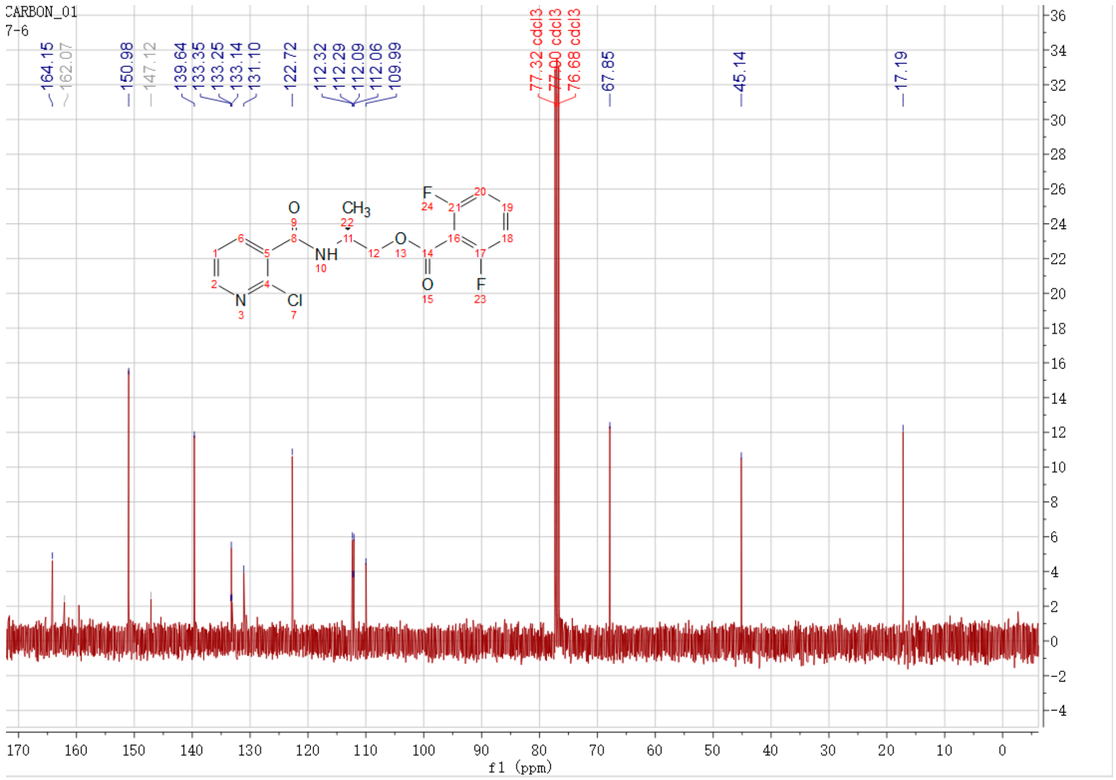

^{13}C NMR of compound 3f

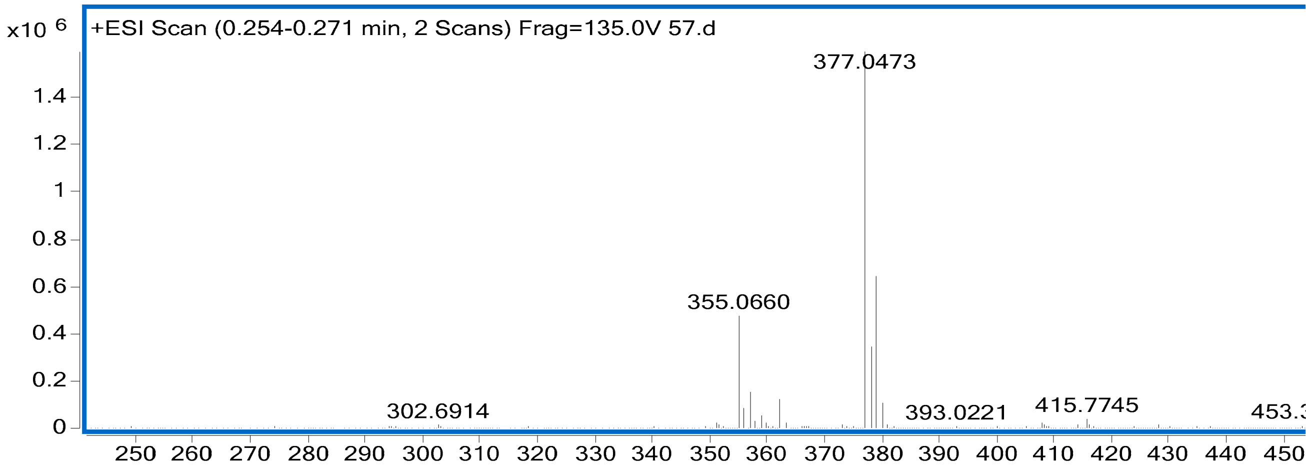

HRMS of compound 3f

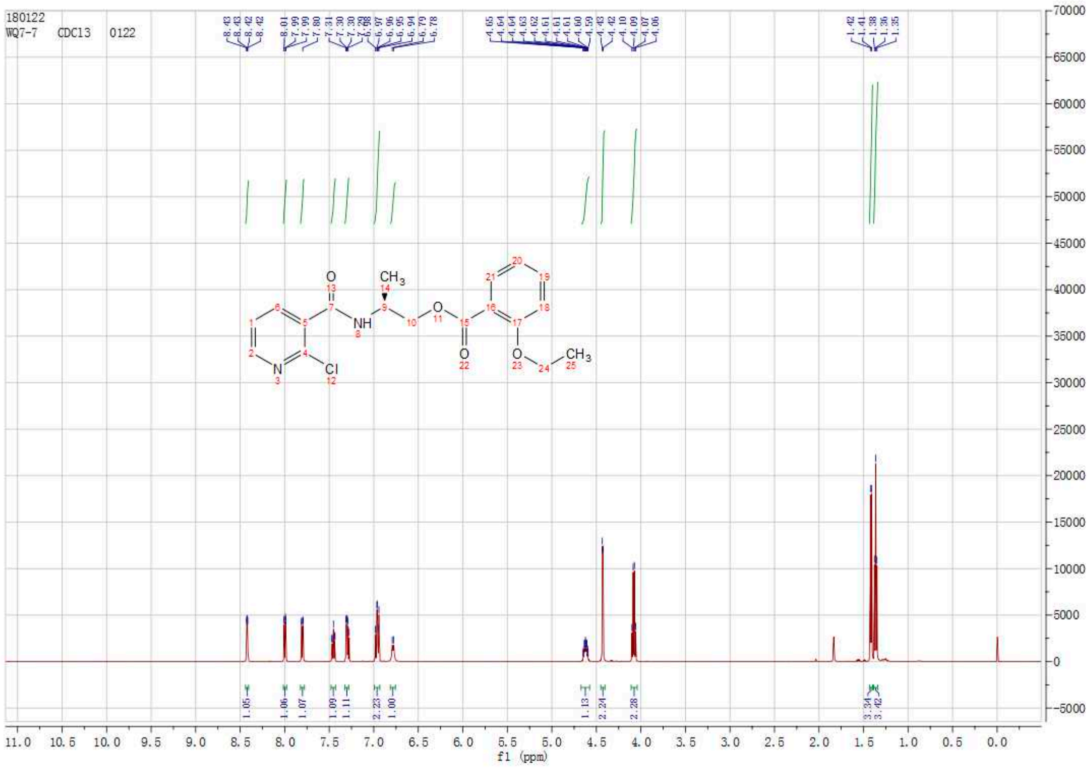

^{1}H NMR of compound 3g

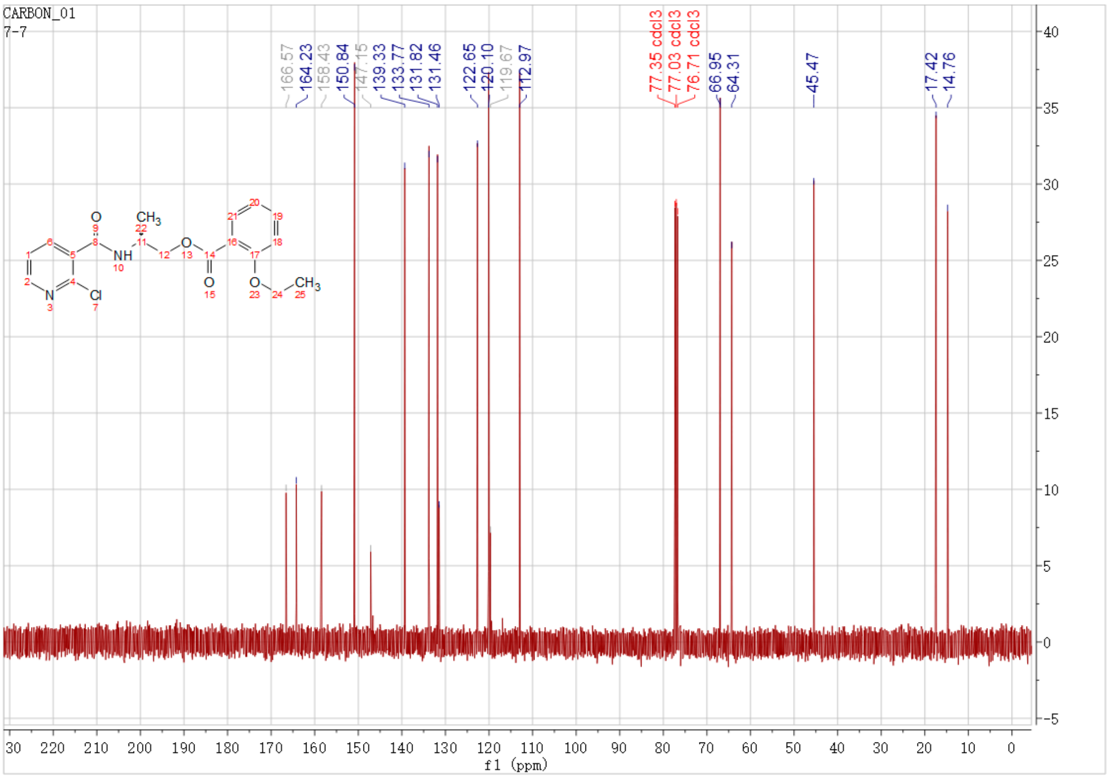

^{13}C NMR of compound 3g

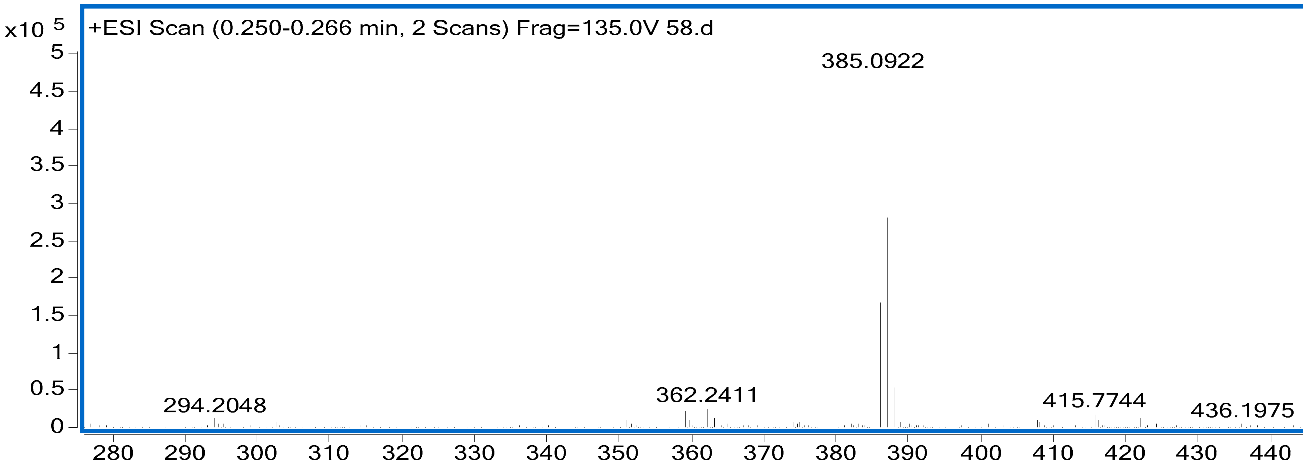

HRMS of compound 3g

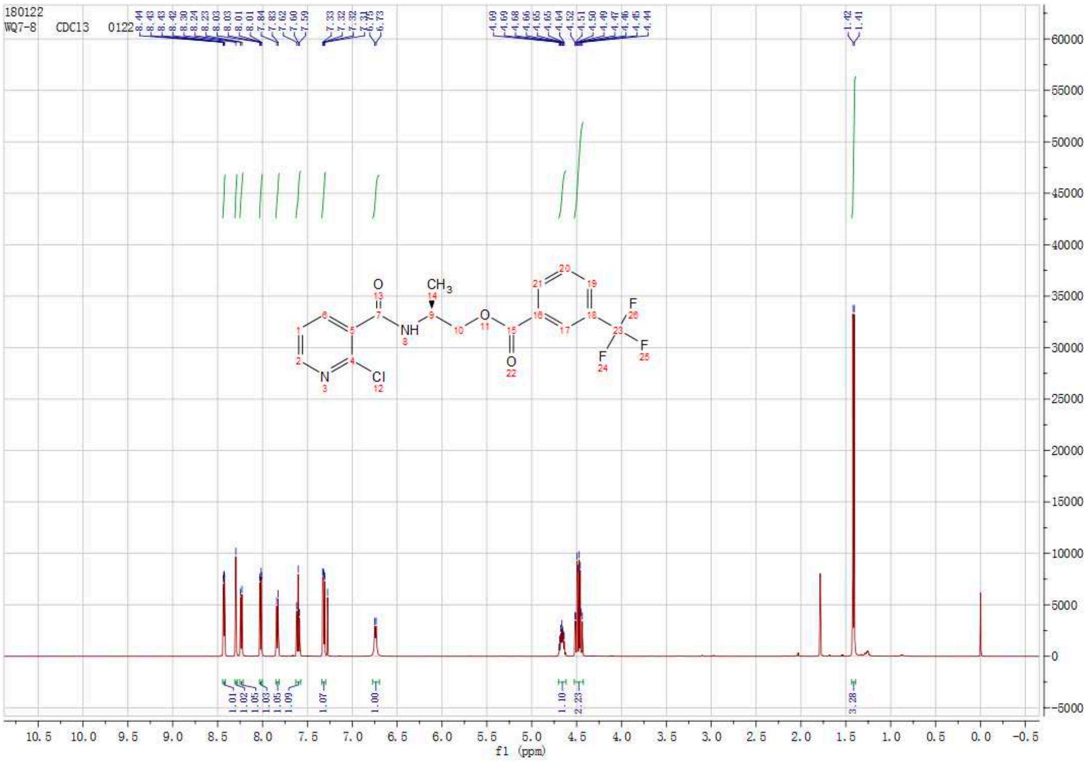

^{1}H NMR of compound 3h

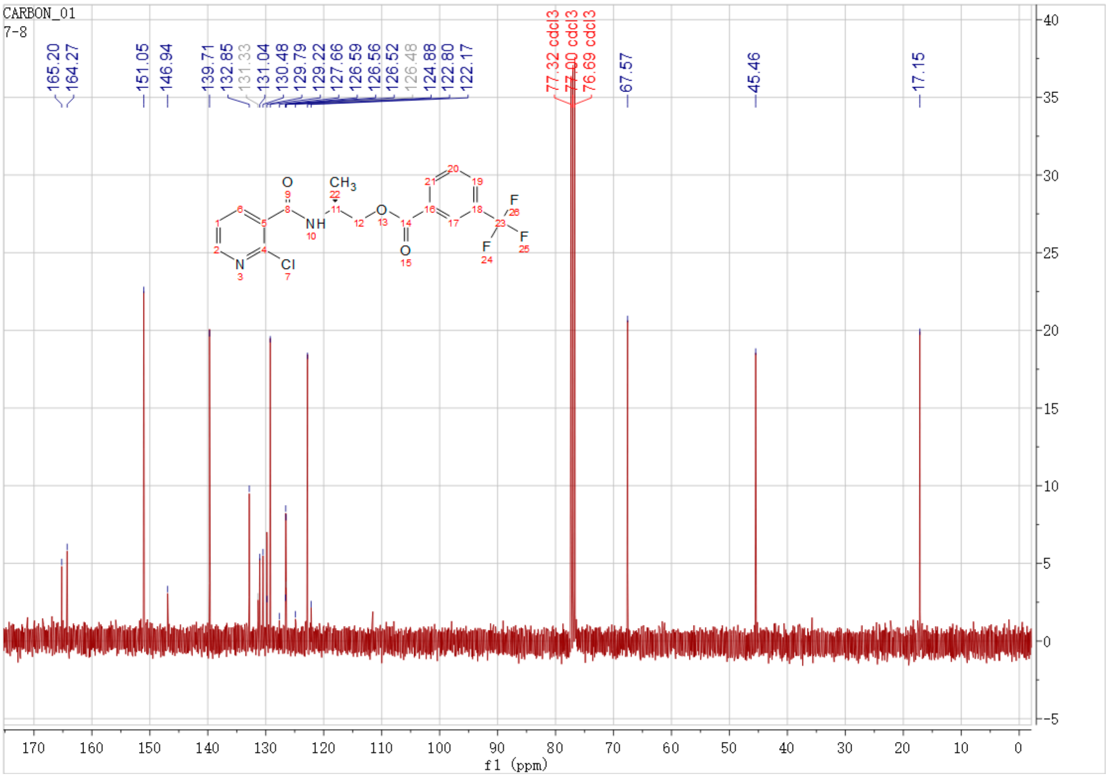

^{13}C NMR of compound 3h

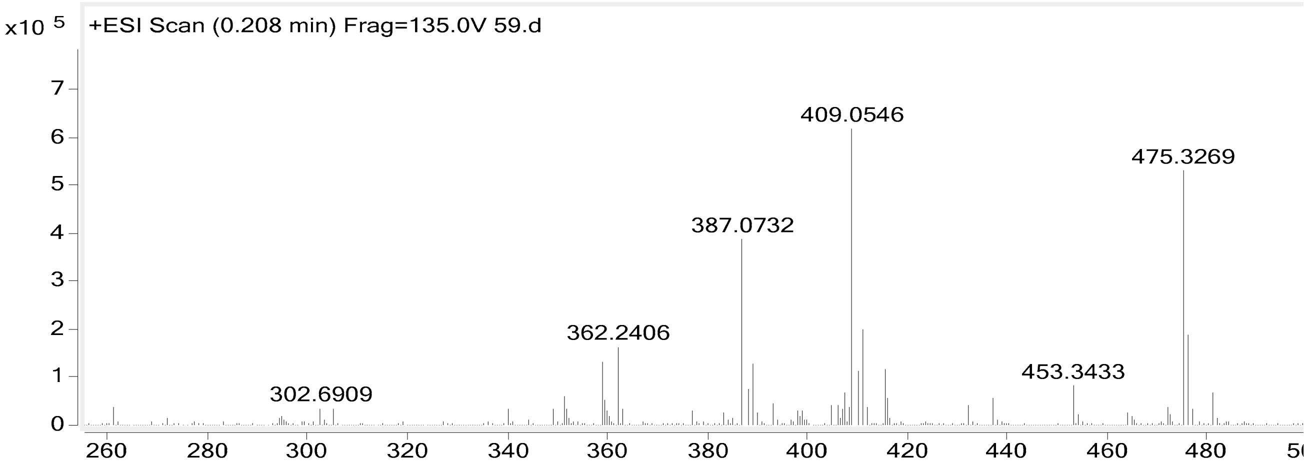

HRMS of compound 3h

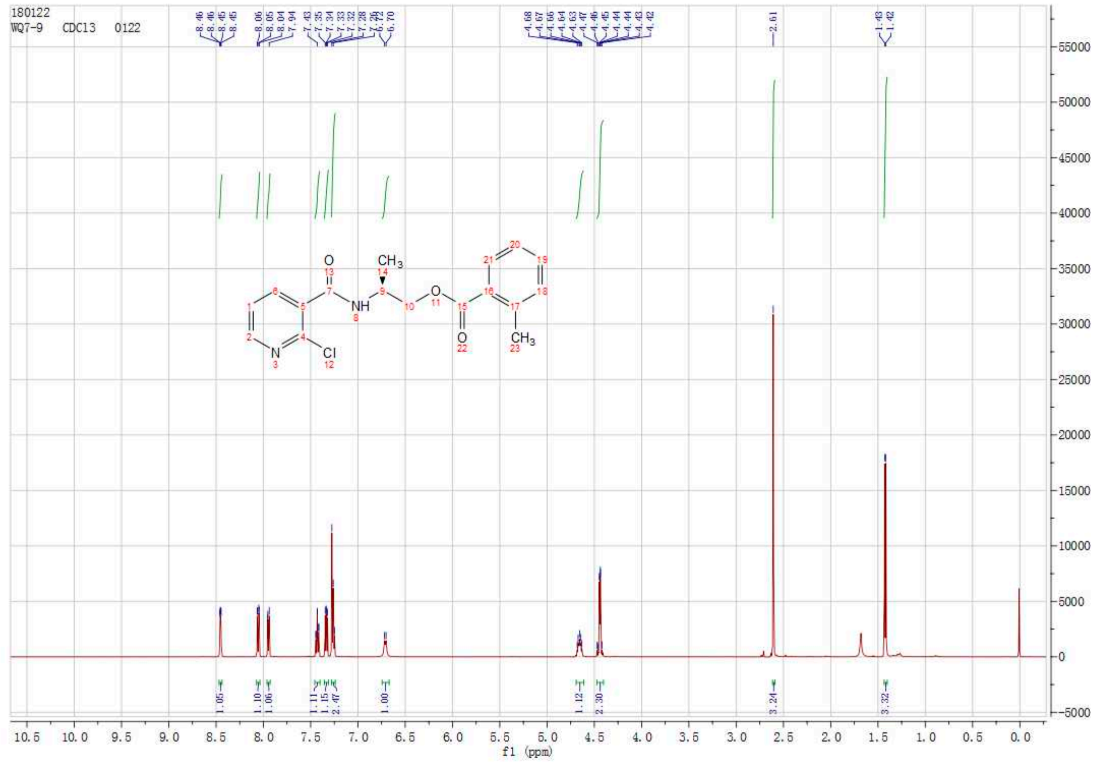

^{1}H NMR of compound 3i

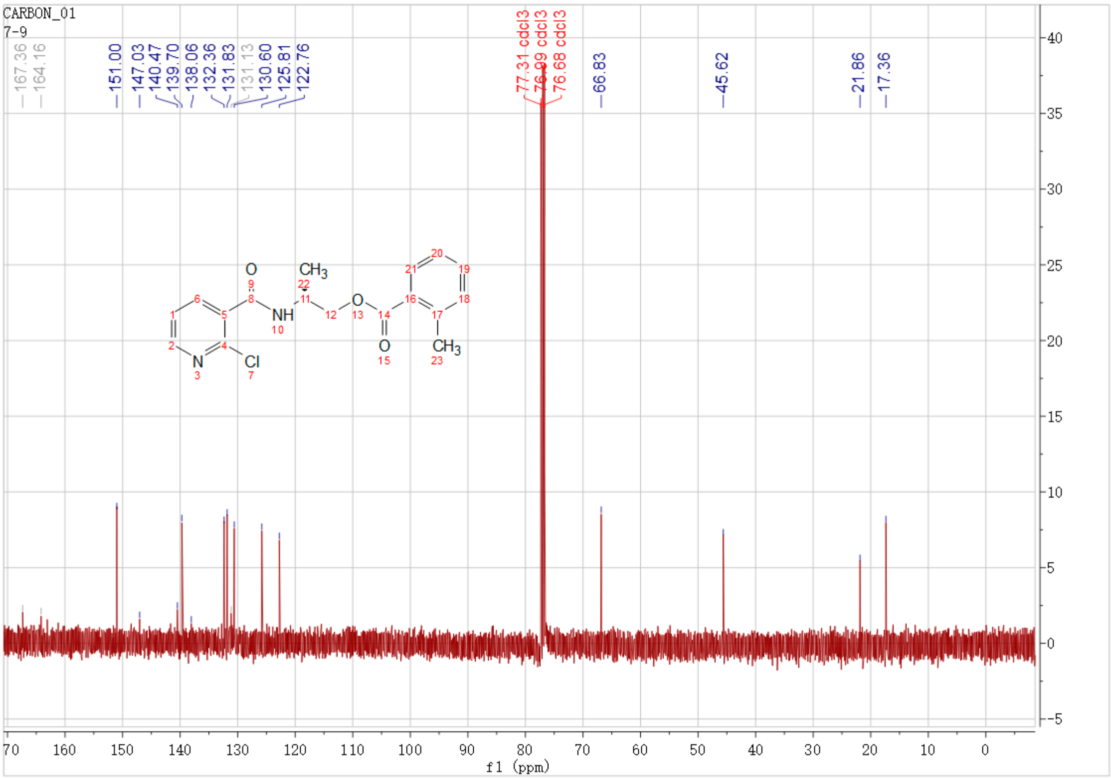

^{13}C NMR of compound 3i

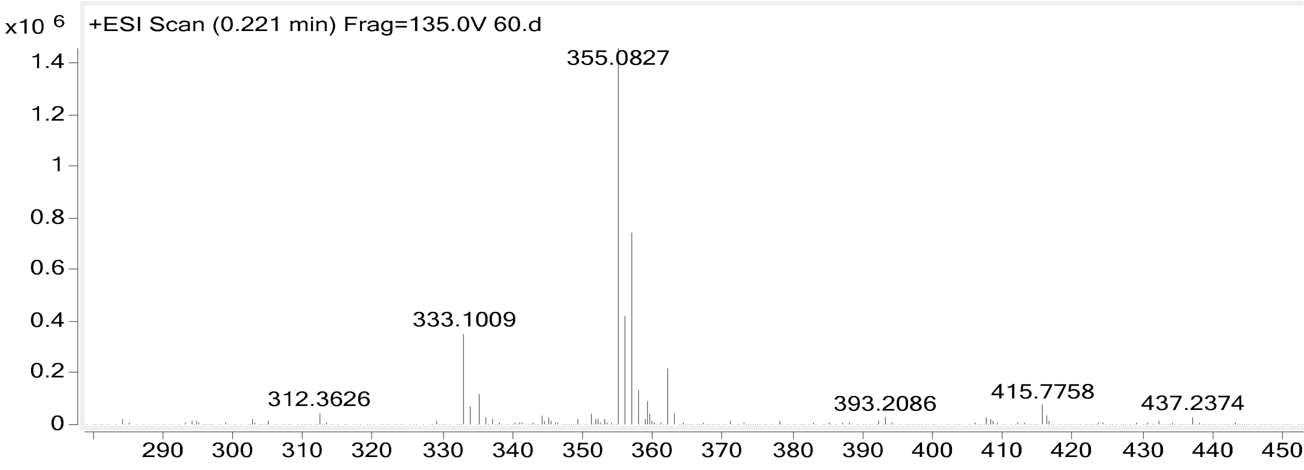

HRMS of compound 3i

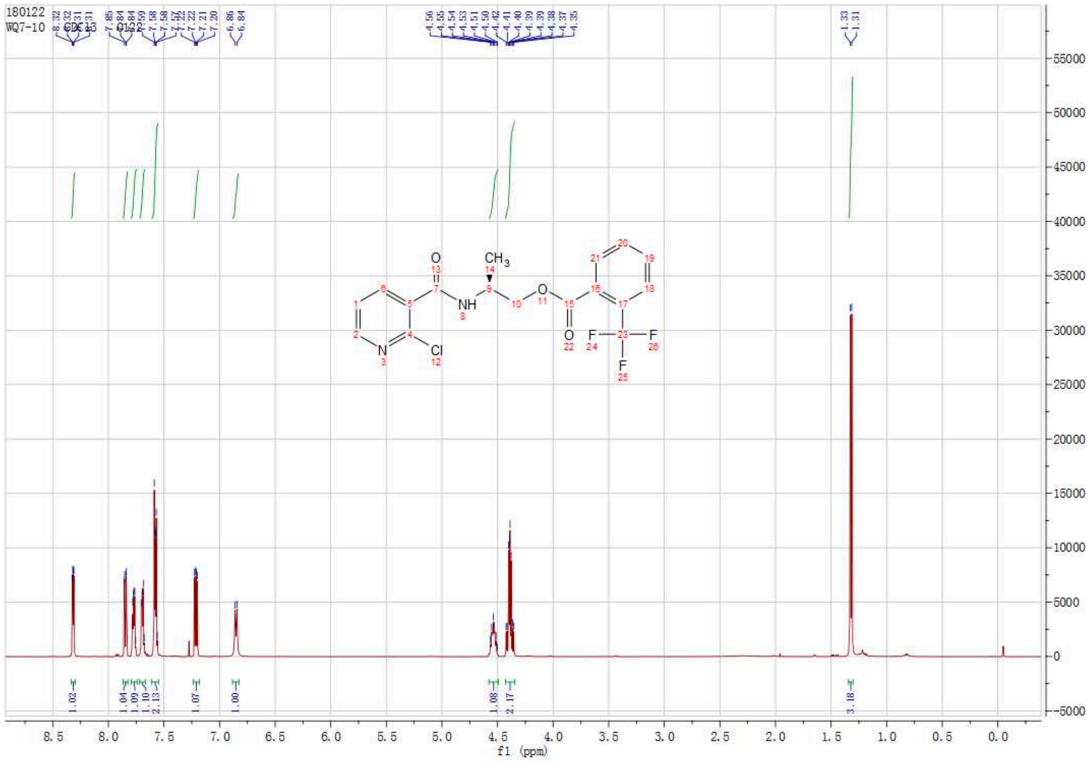

^{1}H NMR of compound 3j

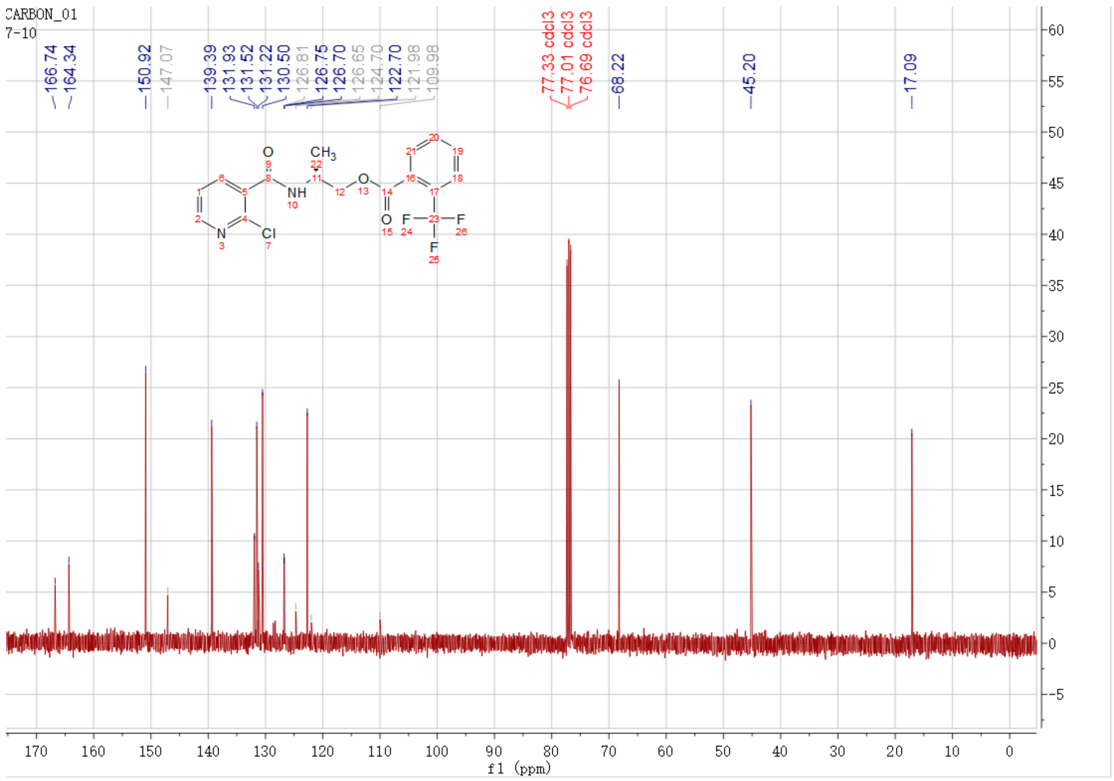

^{13}C NMR of compound 3j

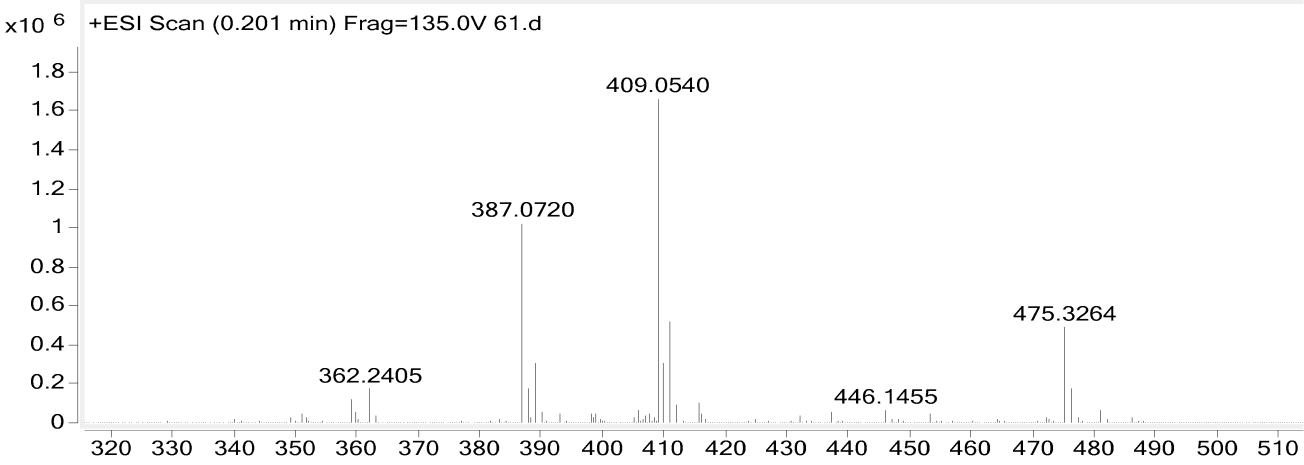

HRMS of compound 3j

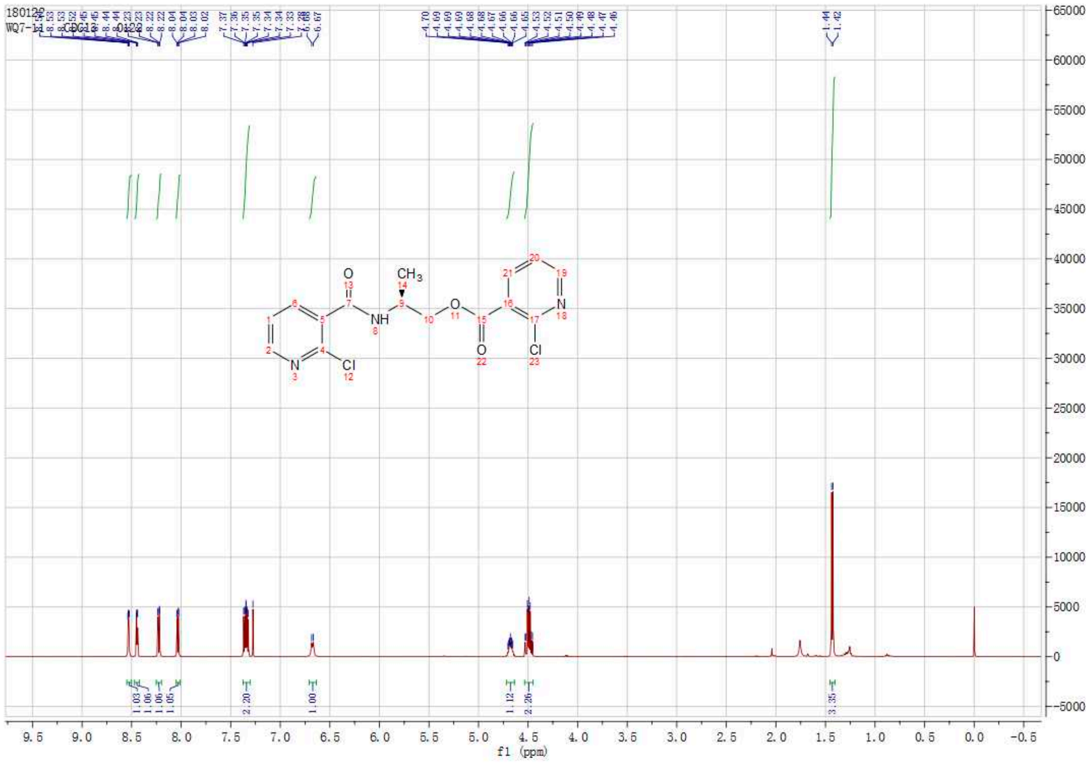

^{1}H NMR of compound 3k

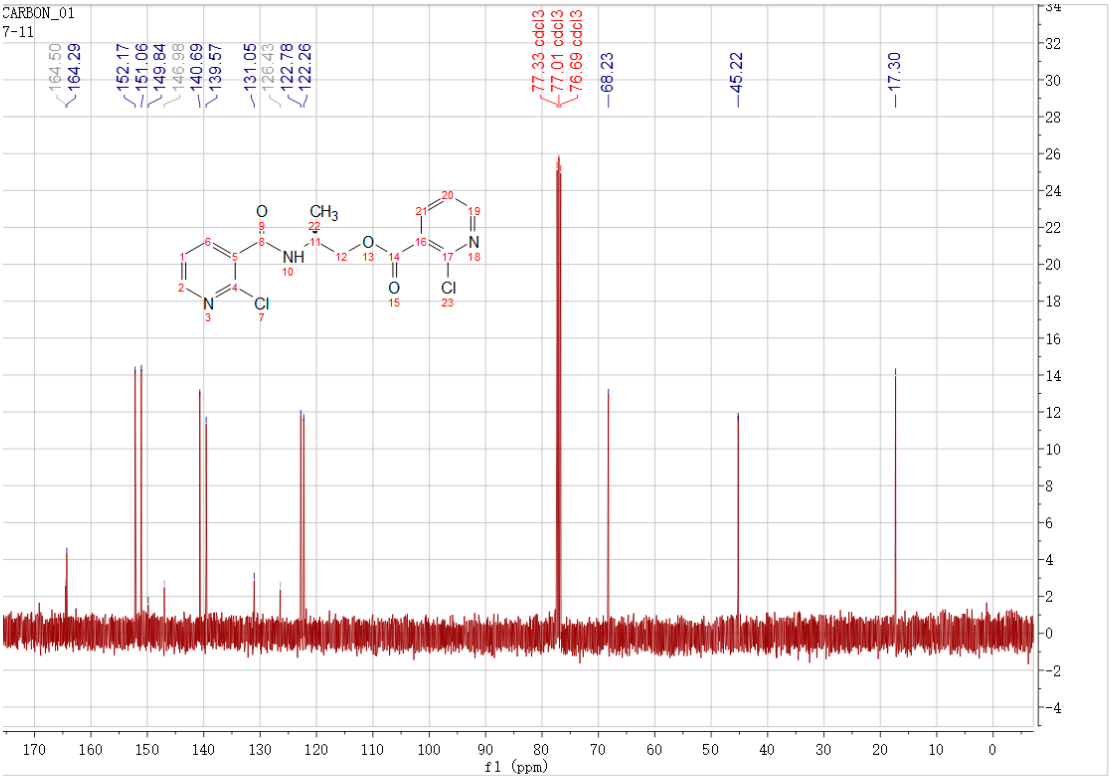

^{13}C NMR of compound 3k

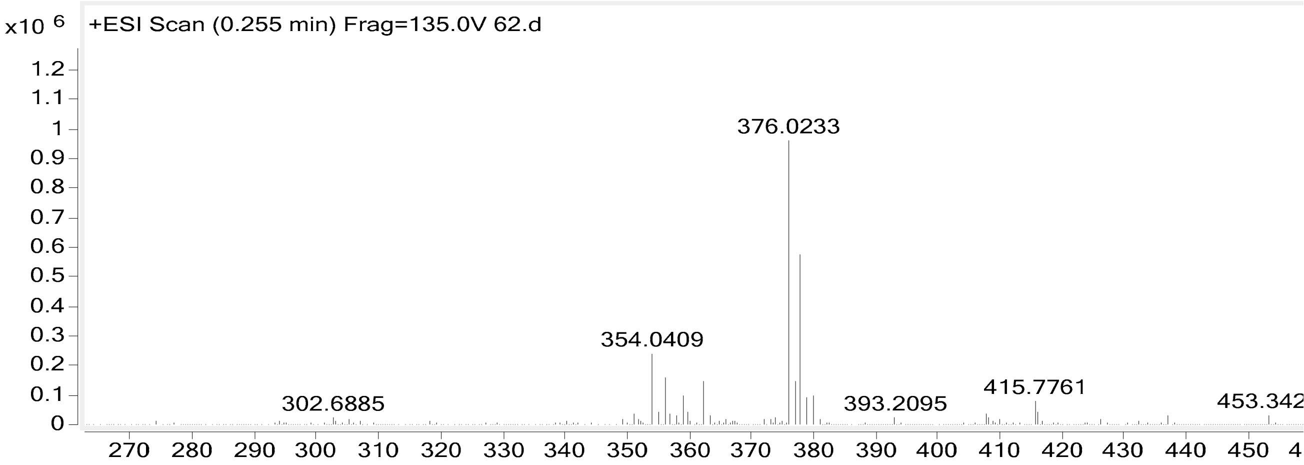

HRMS of compound 3k

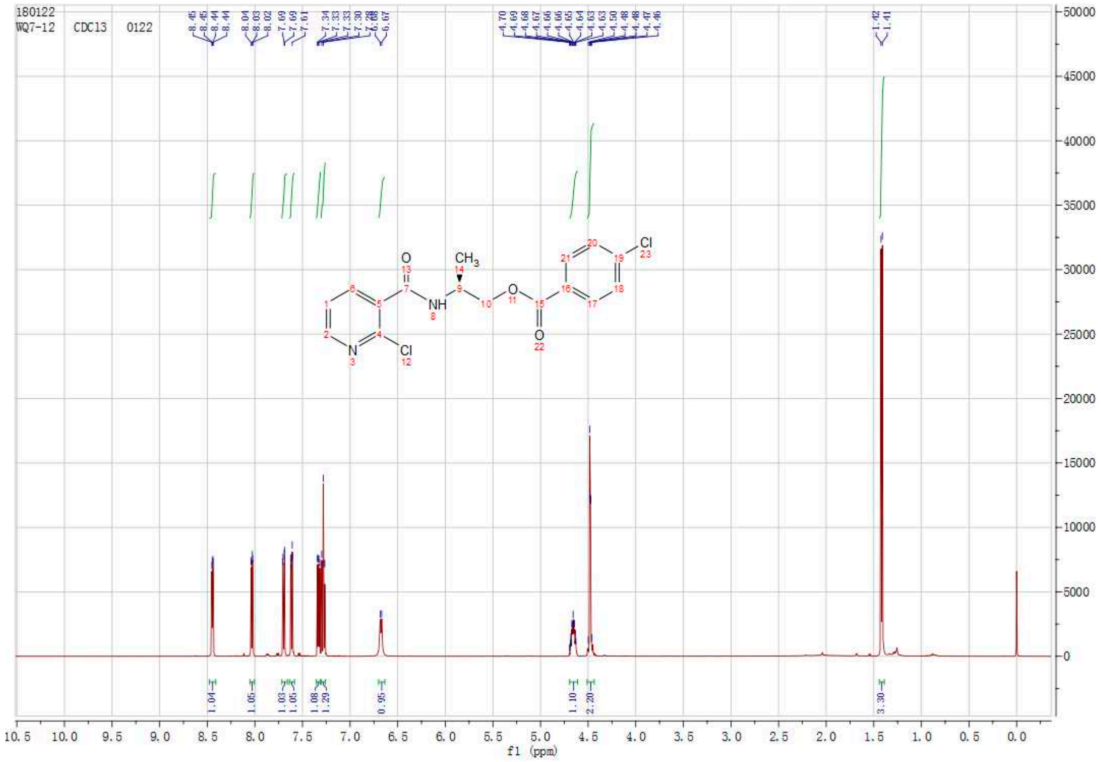

^{1}H NMR of compound 3l

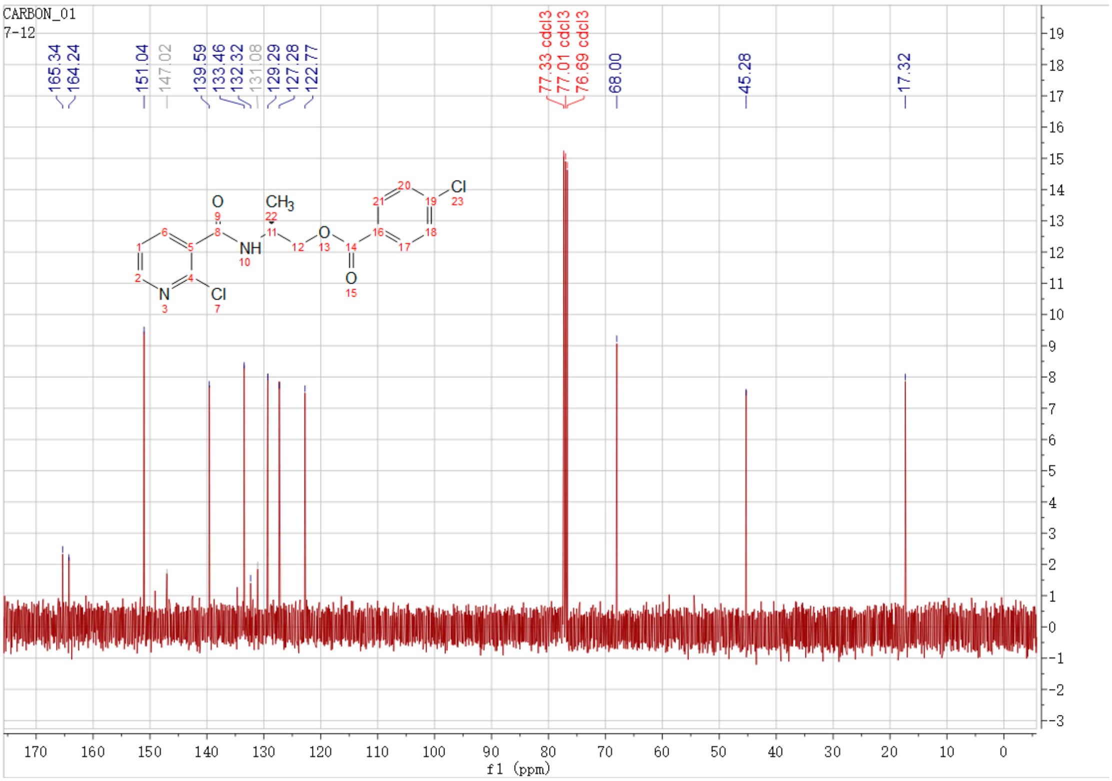

^{13}C NMR of compound 3l

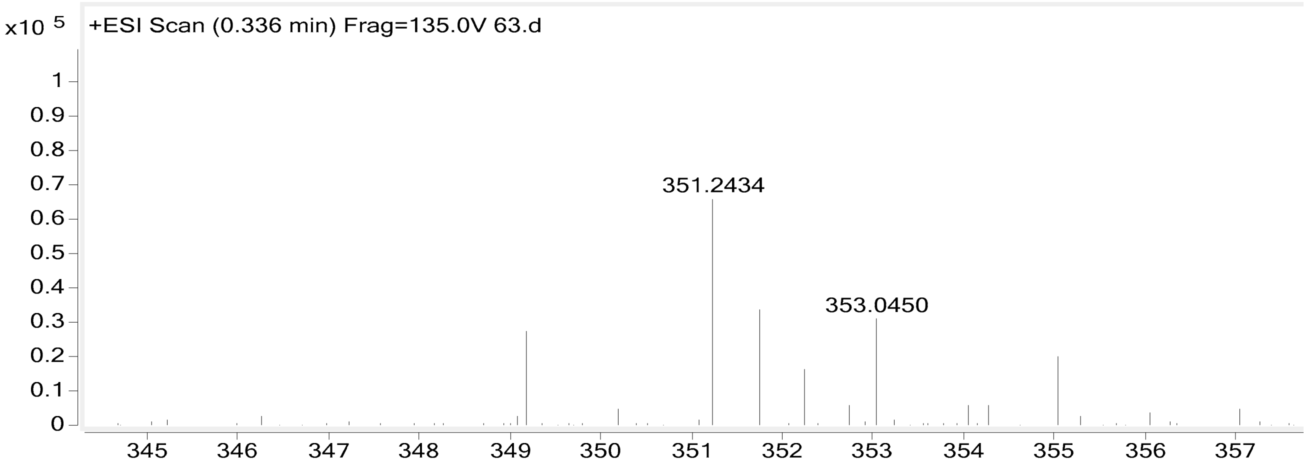

HRMS of compound 3l

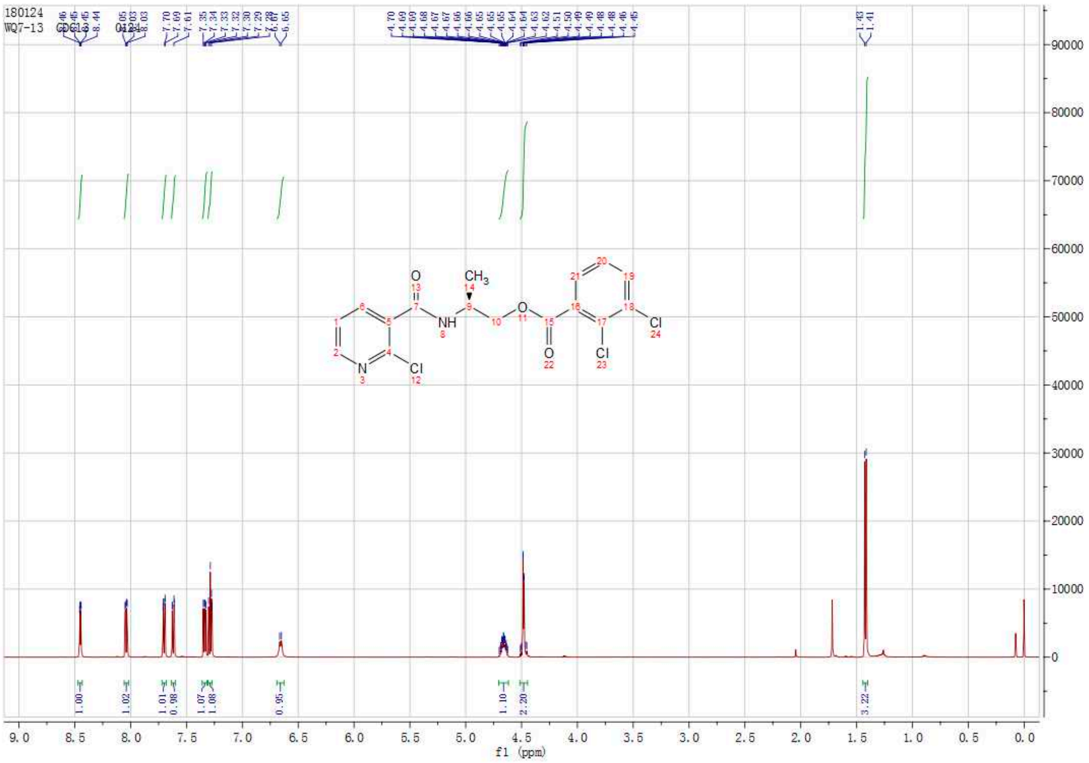

^{1}H NMR of compound 3m

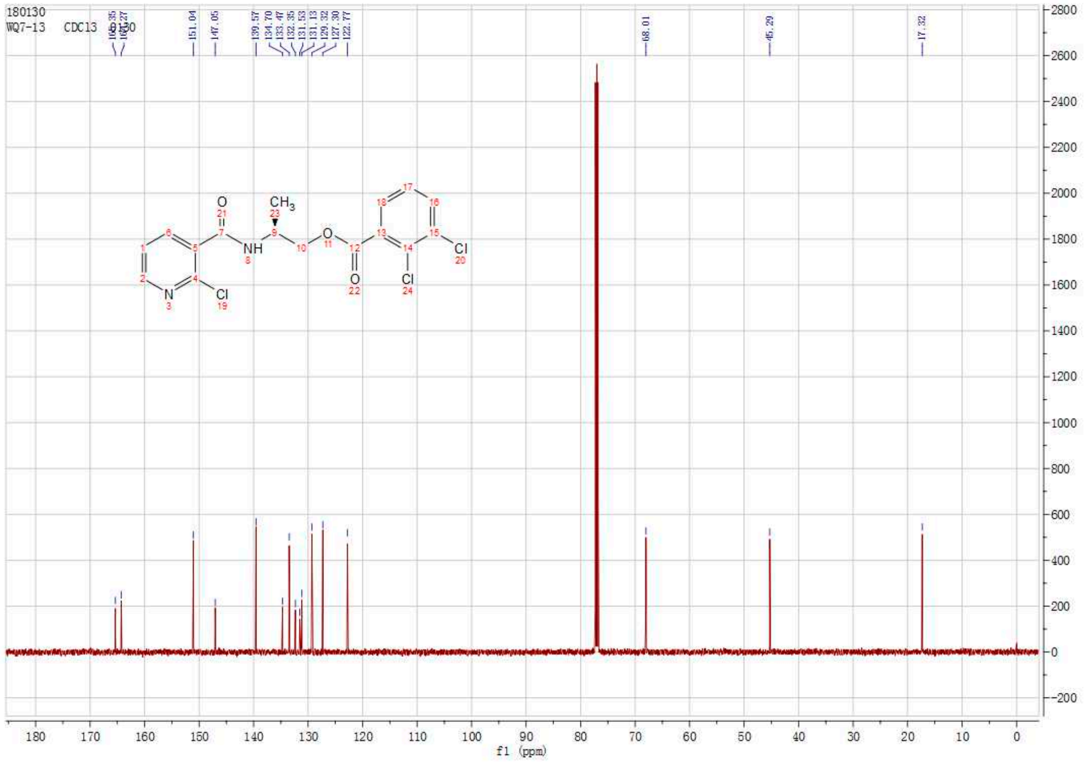

^{13}C NMR of compound 3m

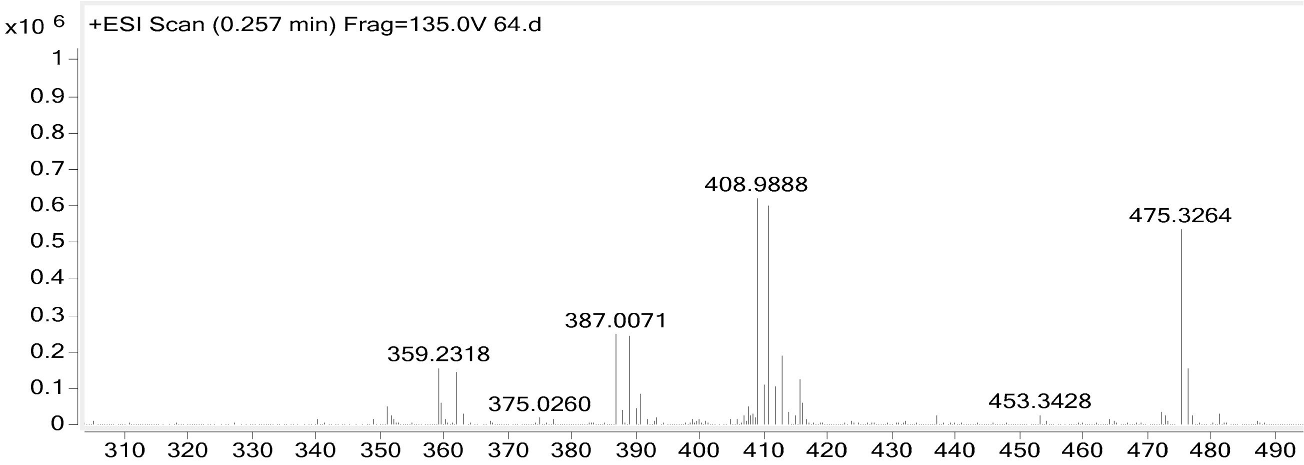

HRMS of compound 3m

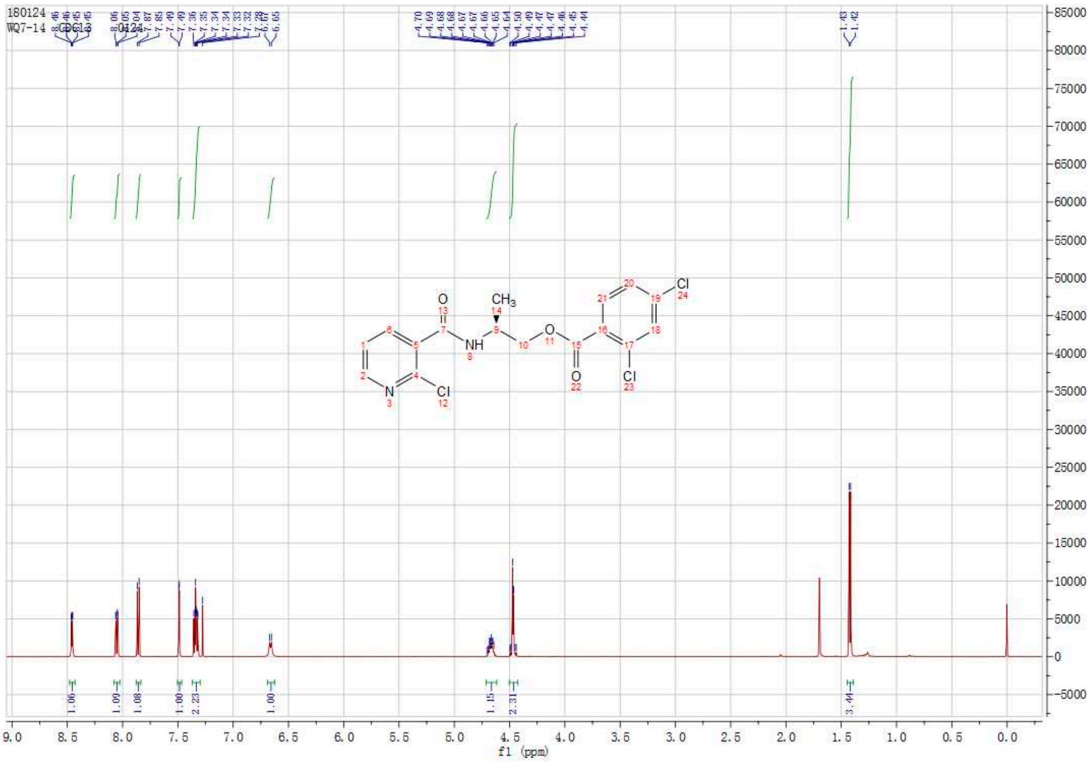

^{1}H NMR of compound 3n

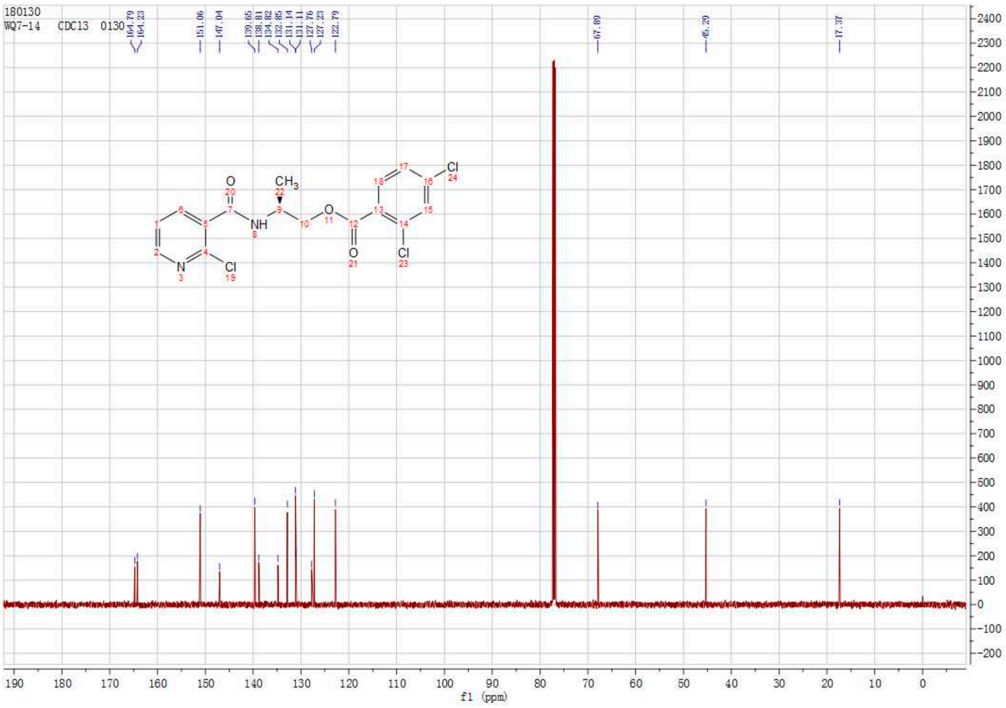

^{13}C NMR of compound 3n

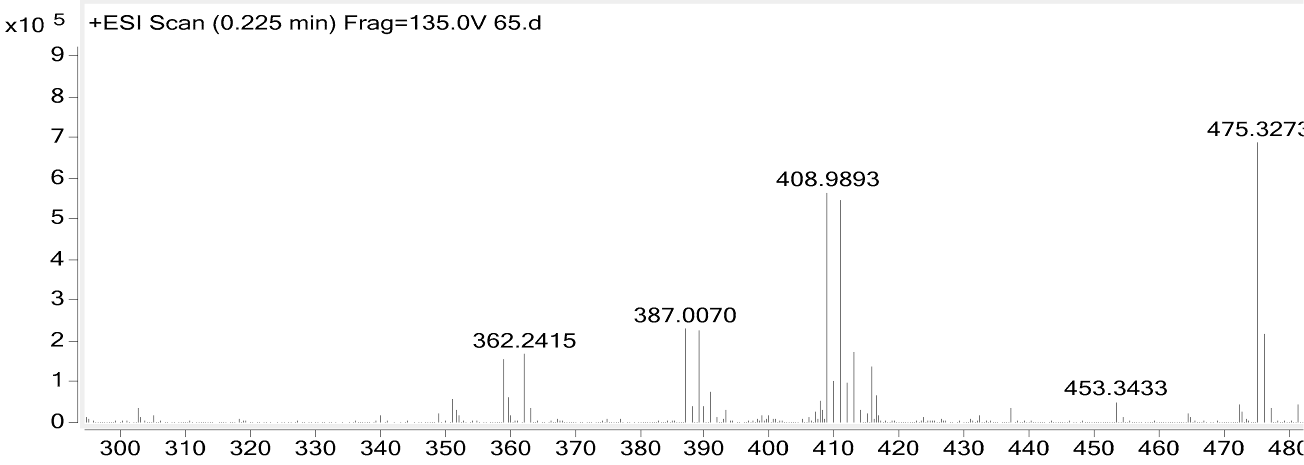

HRMS of compound 3n

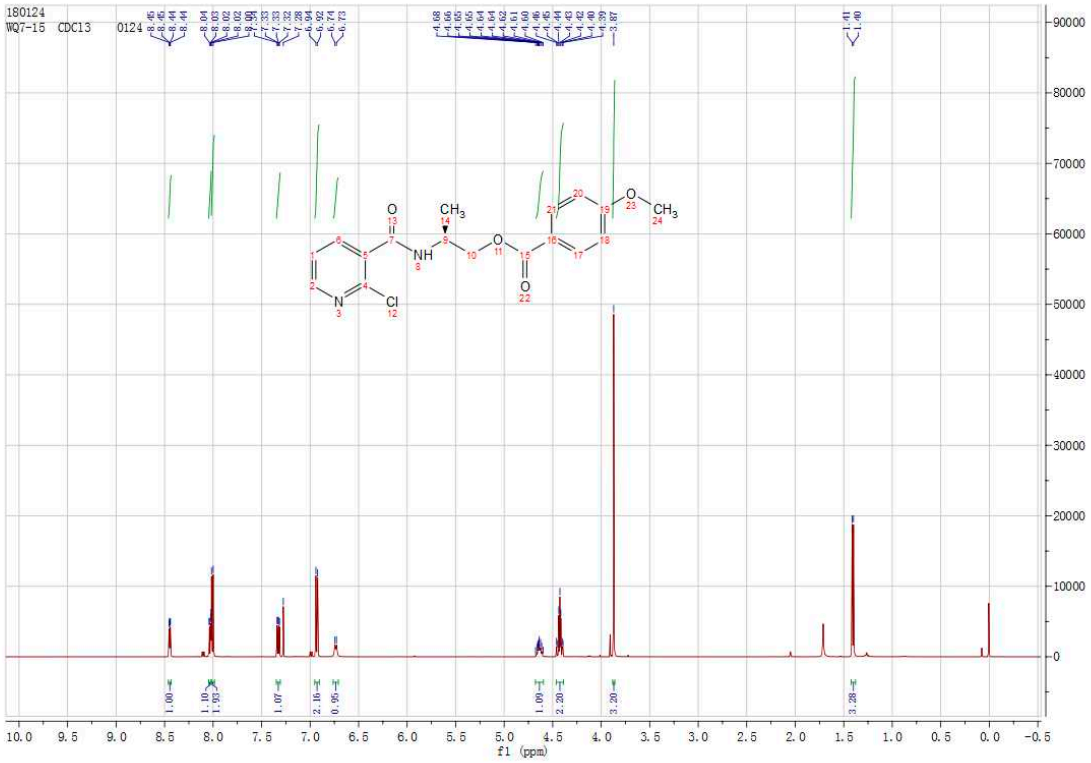

^{1}H NMR of compound 3o

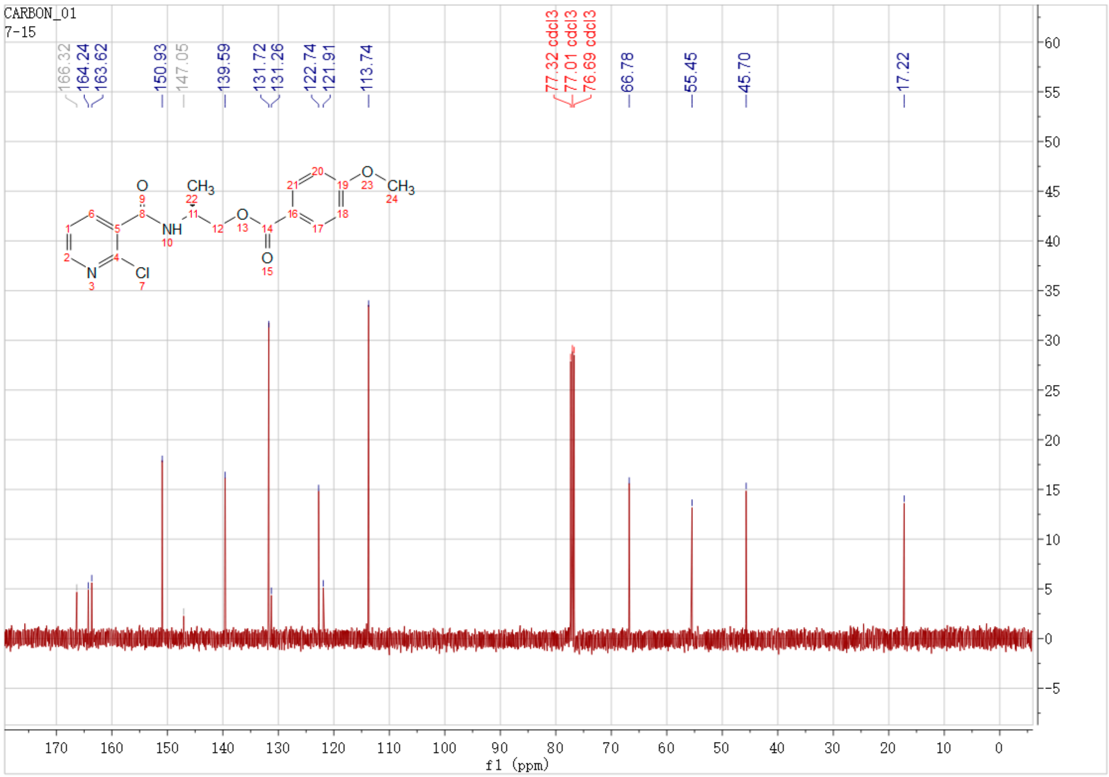

^{13}C NMR of compound 3o

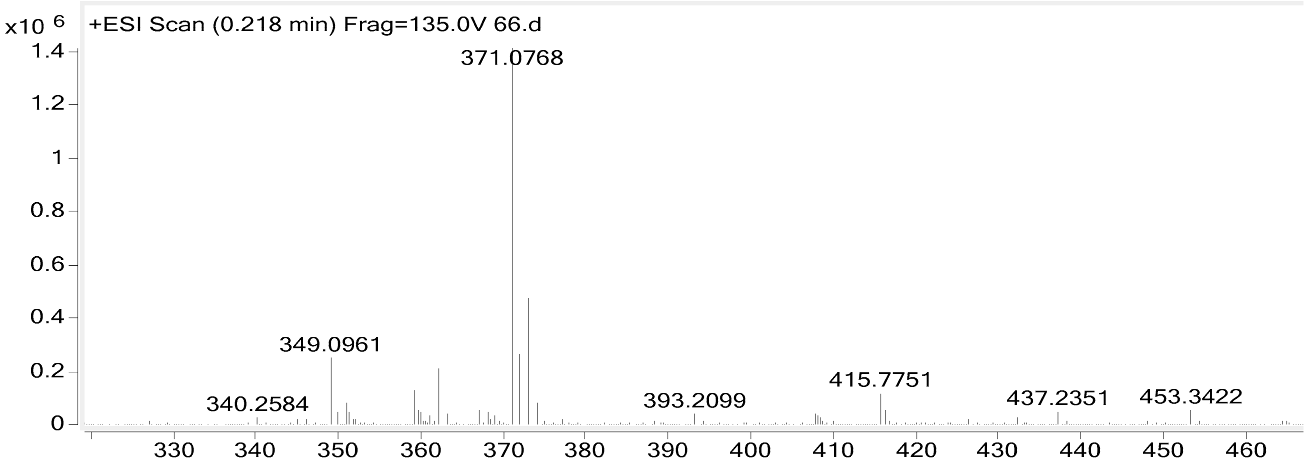

HRMS of compound 3o

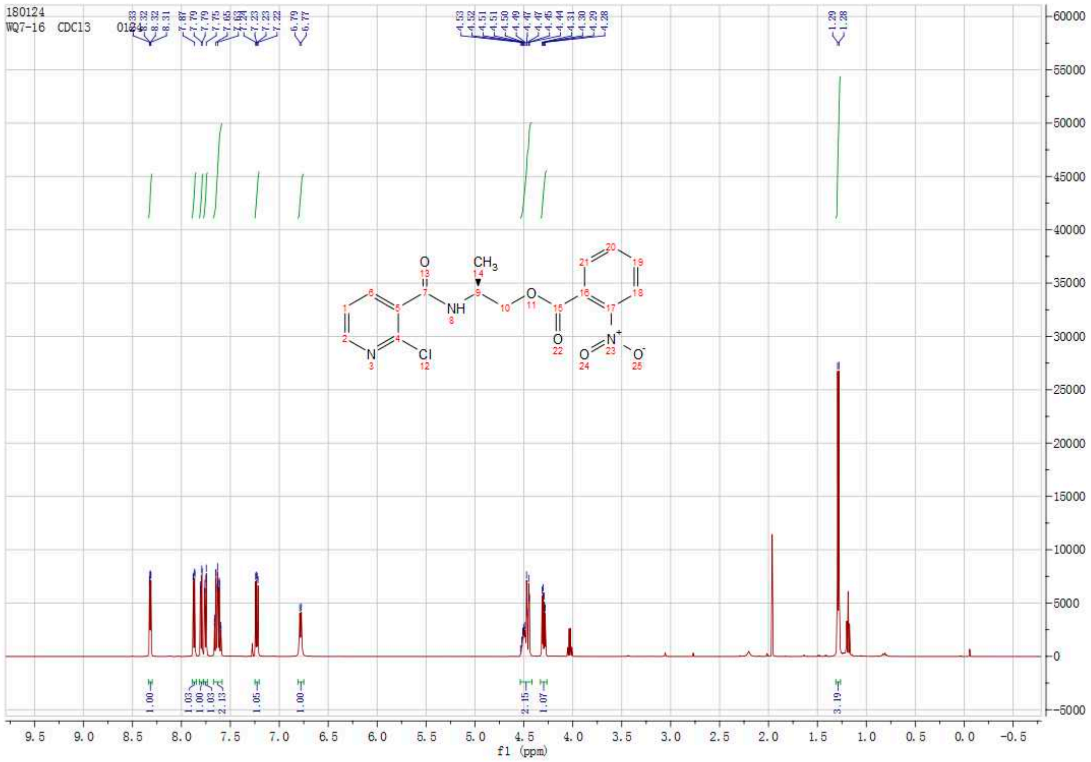

^{1}H NMR of compound 3p

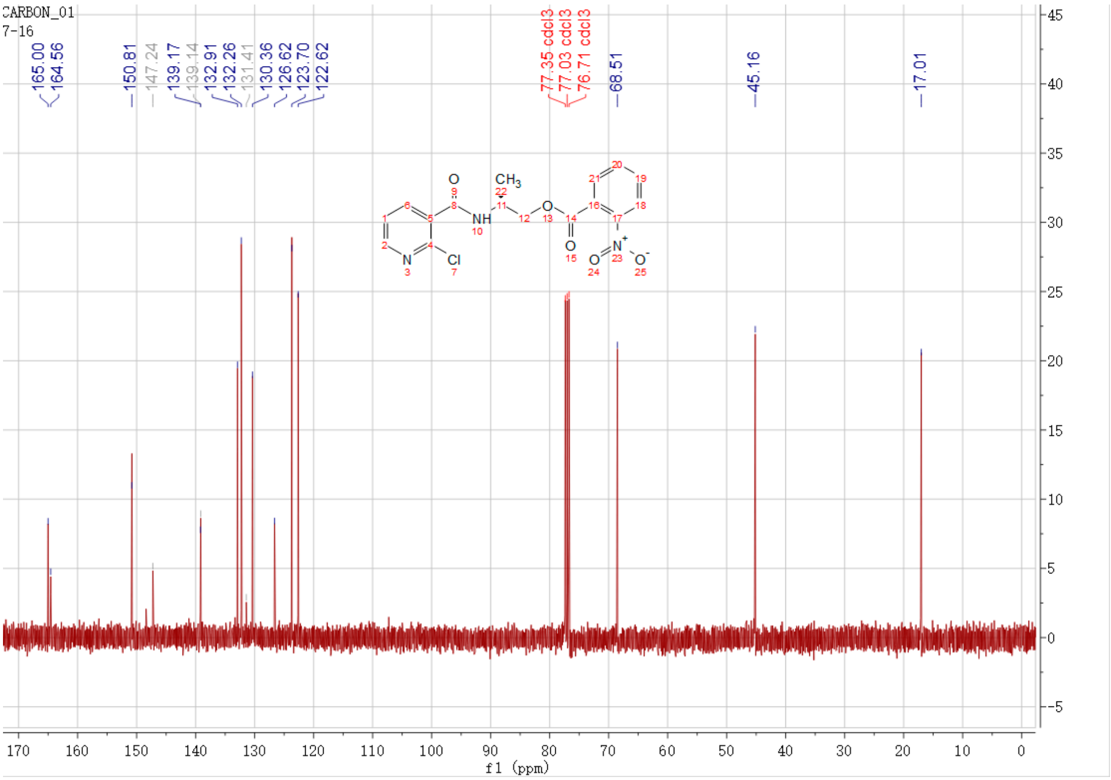

^{13}C NMR of compound 3p

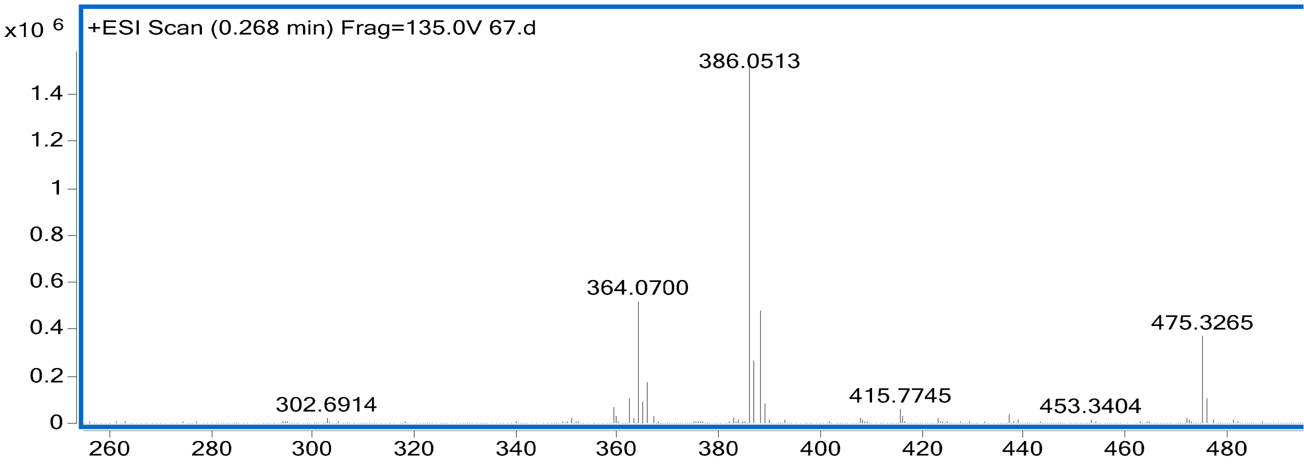

HRMS of compound 3p
